# Biological Barriers to Forest Pest Invasions: A Novel Host Tree Slows Mountain Pine Beetle Range Expansion

**DOI:** 10.1002/ece3.72296

**Published:** 2025-10-16

**Authors:** Evan C. Johnson, Antonia Musso, Catherine Cullingham, Mark A. Lewis

**Affiliations:** ^1^ Naos Marine Laboratories Smithsonian Tropical Research Institute Ancón Panama; ^2^ Biological Sciences University of Alberta Edmonton Alberta Canada; ^3^ Department of Biology Carleton University Ottawa Ontario Canada; ^4^ Department of Mathematics and Statistics University of Victoria Victoria British Columbia Canada; ^5^ Department of Biology University of Victoria Victoria British Columbia Canada

**Keywords:** bark beetles, forest management, forest pest dynamics, invasion, jack pine, mountain pine beetle, pest management, range expansion

## Abstract

Mountain pine beetle breached the Canadian Rocky Mountains—a former geographic barrier—initiating an eastward range expansion that threatens pine forests across North America. However, mountain pine beetle's expansion stalled unexpectedly in eastern Alberta, defying predictions of rapid spread through jack pine, a novel host tree. We investigated the mechanisms behind this slowed spread using an integrated methodology combining helicopter survey data, statistical modeling, simulations, and a consideration of experimental data. While previous hypotheses attributed the slowed spread to lower pine volumes and stem densities in eastern Alberta's forests, our findings indicate that jack pine's inherent phenotypic characteristics—specifically its smaller size, thinner phloem, and lower monoterpene concentrations—are the main factors limiting beetle success. Mountain pine beetle's limited spread is primarily caused by difficulties in locating and successfully attacking jack pine trees, rather than challenges with reproduction or larval survival within jack pine. Jack pine's traits appear to provide natural resistance against mountain pine beetle invasion, suggesting a lower risk of continued eastward spread than previously assumed. However, given the significant implications for forest management policy and the uncertainties inherent in ecological forecasting, we recommend maintaining beetle monitoring programs.

## Graphical abstract

Mountain pine beetle's eastward expansion through Alberta has stalled unexpectedly in jack pine forests, defying predictions of rapid spread. Using helicopter survey data, statistical modeling, and simulations, we demonstrate that this slowed expansion is primarily due to the inherent characteristics of jack pine rather than forest structure differences, with beetles struggling to locate and successfully attack jack pine trees. The unique phenotype of jack pine appears to provide natural resistance against mountain pine beetle invasion, suggesting a lower risk of continued eastward spread than previously assumed.

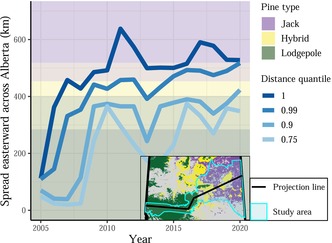

## 1 Introduction

The most recent outbreak of the mountain pine beetle (MPB; *Dendroctonus ponderosae* Hopkins), occurring roughly from 2000 to 2015, was the largest bark beetle outbreak ever recorded (Taylor et al. 2006). Spanning across western North America from Arizona to Yukon, this hyperepidemic killed up to 8 million hectares of pine trees (Meddens et al. 2012), and killed > 50% of merchantable pine in British Columbia alone (British Columbia Government 2016). The outbreak caused economic harm (Corbett et al. 2016; Abbott et al. 2009) and contributed to carbon emissions via wood decomposition (Kurz et al. 2008). The outbreak had numerous impacts on hydrological functioning (Schnorbus 2011; Redding et al. 2008) and forest ecosystems (Dhar et al. 2016, and sources therein). Fire suppression and climate change are believed to be major drivers—MPB thrived due to the weakened defenses of drought‐stressed trees and reduced beetle mortality from milder winters (Carroll et al. 2006; Alfaro et al. 2009; Creeden et al. 2014). Fire suppression led to a buildup of large‐diameter pines, which are favored by MPB (Taylor et al. 2006).

The recent outbreak was so severe that MPB expanded its historic range in a northeasterly direction, reaching northern British Columbia, the Yukon, the Northwest Territories, and most notably, Alberta (Nealis and Cooke 2014). In central British Columbia circa 2005, large numbers of MPB flew above forest canopies—a behavior hypothesized to occur when beetles are abundant and suitable trees are scarce (Carroll and Safranyik 2003; Bleiker 2019). These beetles were carried by windstorms across the Rocky Mountains, overcoming a geographical barrier that had historically confined their range (Safranyik et al. 1989, 1992; Jackson et al. 2008; de la Giroday et al. 2012). There is a nearly contiguous path of pine trees across Canada's boreal zone, so MPB's arrival in Alberta threatened forests spanning to the east coast.

**FIGURE 1:**
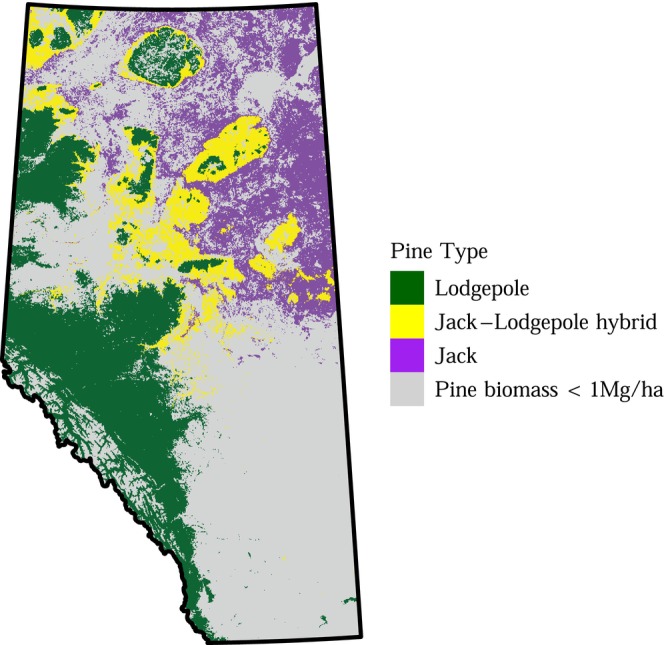
Lodgepole pine inhabits western Alberta, while jack pine inhabits eastern Alberta. There is a large zone in central Alberta where the two species hybridize. The pine species data come from Cullingham et al. (2012) and Burns et al. (2019). The pine biomass data come from Beaudoin et al. (2014).

MPB's ability to spread across Canada is contingent on its ability to spread through a novel host tree, jack pine ( *Pinus banksiana* ). Jack pine inhabits eastern Alberta, whereas lodgepole pine ( *Pinus contorta* )—MPB's primary host throughout the Rocky Mountains and the Pacific Northwest—inhabits western Alberta (Figure 1). Initially, the dominant view was that MPB would rapidly spread through jack pine: a working group of MPB researchers concluded that “There are therefore no known biological impediments to the spread and establishment of the mountain pine beetle through the boreal zone”, and that “The dynamic behavior of beetles on an invasion front, including the rate of spread, will not differ from observed behavior in similar situations within the historic range” (Nealis and Peter 2008).

**FIGURE 2:**
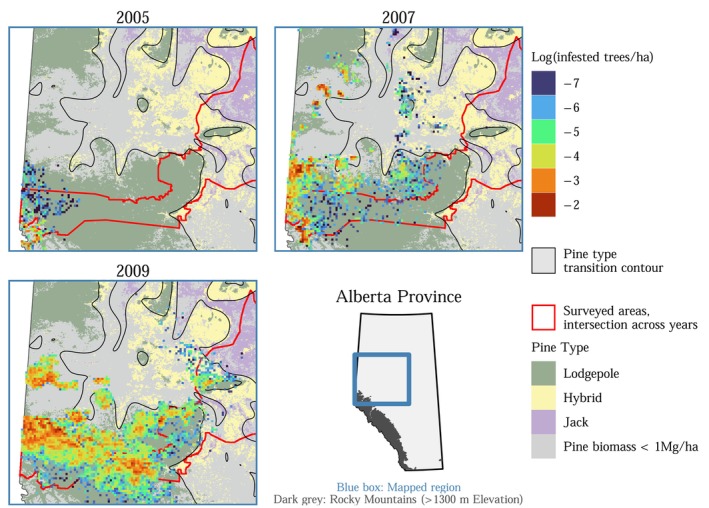
Mountain pine beetle rapidly spread across western Alberta from 2005 to 2009. Infestation data come from Heli‐GPS surveys.

**FIGURE 3:**
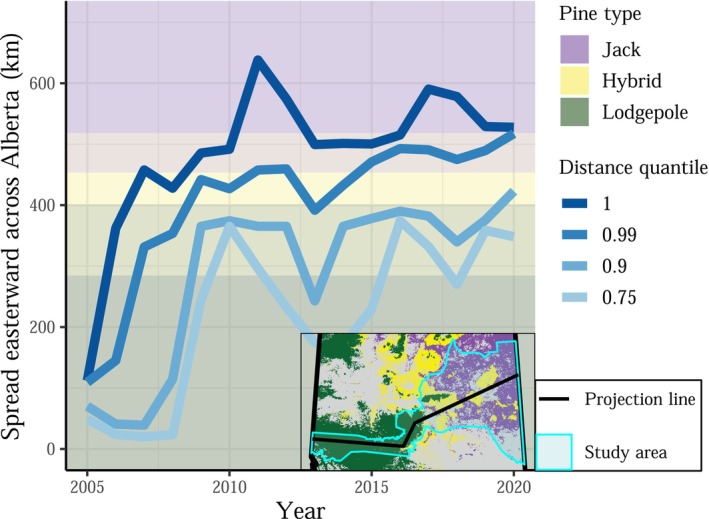
Mountain pine beetle's range expansion slowed down significantly around 2009–2010. Spread distance is summarized by projecting infestations onto a single dimension: a line running through the center of the consistently surveyed area (methods in Section 2.4).

Mountain pine beetle's range expansion took several unexpected turns (Figure 2). Between 2004 and 2006, MPB spread more rapidly than anticipated, quickly reaching areas where lodgepole pine and jack pine hybridize. Most notably, MPB leaped over 220 km eastward in 2006 (Cooke and Carroll 2017). From 2007 to 2009, the spread of MPB continued slightly farther eastward, and large MPB populations developed in lodgepole pine forests around Marten Mountain and directly south of Lesser Slave Lake, areas adjacent to hybrid and jack pine forests. The first successful mountain pine beetle infestation in jack pine, where offspring were produced, was observed in 2009 (Cullingham et al. 2011). However, MPB's range expansion then stagnated. For more than a decade, MPB has been unable to proliferate through the jack pine forests of eastern Alberta (Figure 3).

Understanding MPB's life history is crucial to understanding MPB's decelerating range expansion. Adult beetles synchronously emerge from their natal trees in July or August (Powell and Bentz 2009; Bleiker and Van Hezewijk 2016). During an initial *host finding/acceptance* stage, females rely on a variety of cues (olfactory, gustatory, and tactile) to locate a suitable host tree (Reid 1963; Raffa and Berryman 1982; Raffa 2001); they select a tree by boring into it, but the tree fights back by exuding toxic resin. The pioneering females emit *trans*‐verbenol, an aggregation pheromone which attracts additional beetles (Pitman and Vité 1969; Pureswaran et al. 2000). Arriving males emit *exo*‐brevocomin, an aggregation pheromone that attracts additional females (Rudinsky et al. 1974). These aggregation pheromones, along with attractive host volatiles, lead to a *mass attack* where swarms of beetles concentrate on a single tree (Raffa and Berryman 1983). Once the tree's resin reserves are exhausted, females begin colonization by excavating galleries and laying eggs. Larvae then eat the tree's phloem throughout autumn and overwinter with the help of cryoprotectants (Régnière and Bentz 2007). They metamorphose in the spring or summer, thus completing their univoltine life cycle.

**FIGURE 4:**
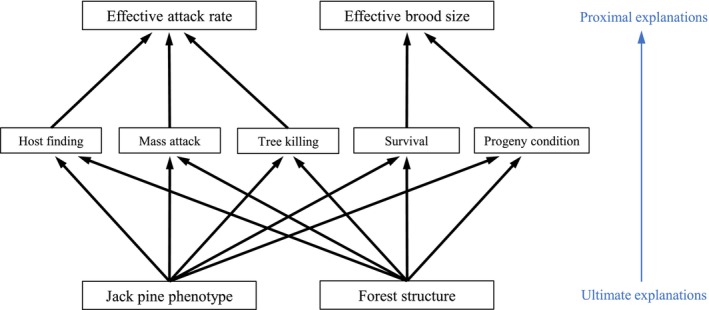
A hierarchy of explanations for MPB's slowed range expansion. The top level consists of model‐based constructs: the *effective attack rate* and the *effective brood size*. These encapsulate the life cycle stages in the middle layer. The bottom layer contains ultimate explanations: species‐level differences between lodgepole pine and jack pine, and environmental/structural differences between the forests of western and eastern Alberta. All arrows indicate positive effects.

A hierarchical framework can be used to organize the numerous explanations for MPB's limited incursion into jack pine forests (Figure 4). At the highest level, there are two proximal explanations: (1) a poor effective attack rate in jack pine forests, and (2) a reduced effective brood size. Here, the concept of an *effective attack rate* encapsulates MPB's ability to find and select hosts, mount mass attacks, and overcome tree defenses. The concept of an effective brood size encapsulates per capita fecundity (oviposition), intraspecific competition among larvae, overwintering mortality, and sub‐lethal effects of climate and tree defenses, which may affect MPB fitness. These are conceptual definitions that each capture several different ecological processes and are represented by multiple model parameters.

At the bottom level of the hierarchy, there are two ultimate explanations for MPB's slow spread: (1) some species‐specific properties of jack pine, and (2) the typical forest structure associated with jack pine in eastern Alberta, that is, reduced canopy closure, smaller trees, and lower connectivity (Burns 1990; Bleiker 2019). The detailed biological mechanisms are conceptualized as arrows between the bottom and middle levels of the hierarchy in Figure 4. For example, MPB may struggle with host‐finding in jack pine forests due to lower levels of *β*‐phellandrene in jack pine (Clark et al. 2014), the only known host monoterpene that attracts beetles in the absence of aggregation pheromones (Miller and Borden 1990). This species‐level property of jack pine—its unique semiochemical profile—could negatively impact MPB's host‐finding abilities, thus negatively affecting the effective attack rate.

We focus on two key contrasts—effective attack rate versus effective brood size, and pine species identity versus forest structure—because they are amenable to analysis with models. Solving the puzzle of MPB's slowed range expansion requires an integrative approach that considers all types of evidence. Thus, we consider (1) bolt experiments, where MPB individuals are introduced into cross‐cut segments of tree trunks; (2) statistical models, which aim to associate MPB demography with various factors, including pine species identity; and (3) circumstantial evidence, that is, clues from small‐scale studies that focus on a single aspect of MPB biology—typically behavior or pheromone‐based communication.

Bolt experiments are crucial for determining the effective brood sizes of MPB in lodgepole pine and jack pine. Adult beetles are introduced to bolts (i.e., a short log section) either by inserting harvested beetles into pre‐drilled holes in the bark (e.g., Rosenberger et al. 2017), or by catalyzing a mass attack by placing bolts next to mass‐attacked trees (e.g., Burke and Carroll 2016), or releasing harvested beetles next to the lower bole (e.g., Musso 2023). Typically, the bolts are held in cold storage to mimic the overwintering period, and later warmed to room temperature, allowing beetle development to continue. Beetle performance is measured using several metrics, including under‐the‐bark brood size, emergence density (per unit tree surface area), beetle body size, and sex ratio. Large beetles have higher fecundity and can fly farther (Safranyik and Carroll 2006; Graf et al. 2012; Evenden et al. 2014). The sex ratio is relevant because a higher proportion of females suggests some degree of overwintering stress, since females are the more robust sex; this is evidenced by a higher proportion of males emerging from trees with thinner phloem (Cole et al. 1976).

Statistical models are essential for assessing the effective attack rate in jack pine, particularly because field experiments for this purpose are exceptionally work‐intensive. Although researchers can induce mass attacks on bolts (see the previous paragraph), experimentally inducing the entire progression of beetle attacks (including host‐finding, host‐selection, and mass attacks) on living trees is much more laborious. Models remain our primary tool for assessing jack pine's impact at the landscape level—an area where research is critically lacking (Bleiker 2019). To date, the only modeling study that utilized pine species as a covariate found a strong negative association between MPB infestations and jack pine (Srivastava and Carroll 2023).

Here, we take an integrative approach, utilizing both models and bolt experiments to assess the effective attack rate and effective brood size in jack pine. First, we analyze a semi‐mechanistic model of MPB dynamics, parameterized with extensive survey data from the government of Alberta. The effective attack rate is low in jack pine, but the effective brood size cannot be precisely estimated due to the small number of infestations in jack pine. However, bolt experiments indicate that the effective brood sizes are similar in lodgepole and jack pine. Next, we analyze a second model in which the effective attack rate depends on pine ancestry and pine volume. Pine ancestry is found to have a larger effect on spread rate, indicating that MPB is responding to some quality of the jack pine, rather than just the forest structure of eastern Alberta. Finally, using circumstantial evidence, we propose biological mechanisms that could explain these observed patterns.

## 2 Methods

### 2.1 Data

The severity and locations of MPB infestations were determined with Heli‐GPS surveys and ground surveys. If a tree is killed by MPB, its needles will turn rust‐red in the summer or autumn of the following year. The Alberta Ministry of Forestry and Parks uses helicopters to find these *red‐topped trees*, recording both the number of trees and GPS coordinates. Field crews go to the locations of red‐topped trees and search for nearby *green‐attack trees*, trees that are infested but not yet displaying red needles. These trees are “sanitized” (i.e., cut, then burned or chipped) to prevent the proliferation and spread of MPB. Heli‐GPS surveys are a high‐quality data source, accurately counting the number of infestations (within +10 trees) for 92% of infestation clusters (Nelson et al. 2006) and a positional accuracy of +30 meters (Government of Alberta 2016). Ground surveys are even more accurate, with a 98.5% detection rate within the surveyed area (Bleiker 2019).

Our models utilize several other data sources. Estimates of pine volume in the Extended Alberta Vegetation Inventory (AVIE; A.B. Ministry of Agriculture, Forestry and Rural Economic Development 2022) were obtained through data‐sharing agreements with forestry companies. Predicted pine ancestry values were obtained using the methodology of Cullingham et al. (2012) and Burns et al. (2019). The authors used a genetic microchip to analyze pine DNA samples and processed this data through the STRUCTURE software program (Pritchard et al. 2000). The software calculated *Q*‐values representing the posterior means of genetic admixture proportions—the estimated fraction of each individual tree's genome inherited from ancestral populations of pure lodgepole pine versus pure jack pine. *Q*‐values range from Q=0 (pure jack pine ancestry) to Q=1 (pure lodgepole pine ancestry), with intermediate values indicating hybridization between the species. Cullingham et al. used environmental variables and logistic regression to predict *Q*‐values across their study area. We applied their environmental regression model to finer‐scale data to predict *Q*‐values across Alberta at 1 km^2^ resolution (compared to the original 10 km^2^ resolution). Throughout the paper we designate jack pine forest as Q<0.1, hybrid forest as 0.1≤Q<0.9, and lodgepole forest as Q≥0.9. These *Q*‐values are expected to correlate with the species‐level characteristics discussed later in the paper.

#### 2.1.1 Data Preparation

The raw Heli‐GPS and ground survey data contained GPS coordinates for red‐topped or sanitized green‐attack trees, respectively. We rasterized this point data into 1 × 1 km pixels. Much finer resolutions would have been computationally prohibitive, given the extensive size of our study area.

The total number of infested trees in pixel x and year t, denoted $I_{t}\left(x\right)$, was calculated as

(1) $$I_{t}\left(x\right)=m_{t}\left(x\right)+r_{t+1}\left(x\right),$$

where $m_{t}\left(x\right)$ is the number of sanitized green‐attack trees in year t, and $r_{t+1}\left(x\right)$ is the number of red‐topped trees in year t+1. Our goal was to predict next year's infestation locations and severity from current year data. Since $I_{t}\left(x\right)$ includes sanitized trees that cannot cause future infestations, we defined a modified predictor variable: $I_{t}^{*}\left(x\right)=I_{t}\left(x\right)-m_{t}\left(x\right)=r_{t+1}\left(x\right)$.

Several steps were taken to prepare the data for model‐fitting. Pixels representing pine volume less than 1 m^3^km^−2^ were removed, as infestations are highly unlikely in these locations; in the model, any beetles dispersing to these pixels are assumed to die. To mitigate the risk of overly narrow credible intervals caused by conditionally non‐independent data (i.e., spatially autocorrelated residuals), we thinned the dataset at a 3 km scale by selecting only the intersection of every third column and row of the data rasters. This decision was informed by patterns of spatially autocorrelated residuals from a model that was fitted with all pixels (see Appendix S1.1). We tested an alternative approach using a Gaussian process latent variable to handle the spatial autocorrelation. This gave similar results as the spatial thinning, but required 5 × 5 km pixels to remain computationally tractable. We therefore chose to proceed with the spatial thinning approach for our final analysis (see Appendix S1.2 for alternative model results).

**FIGURE 5:**
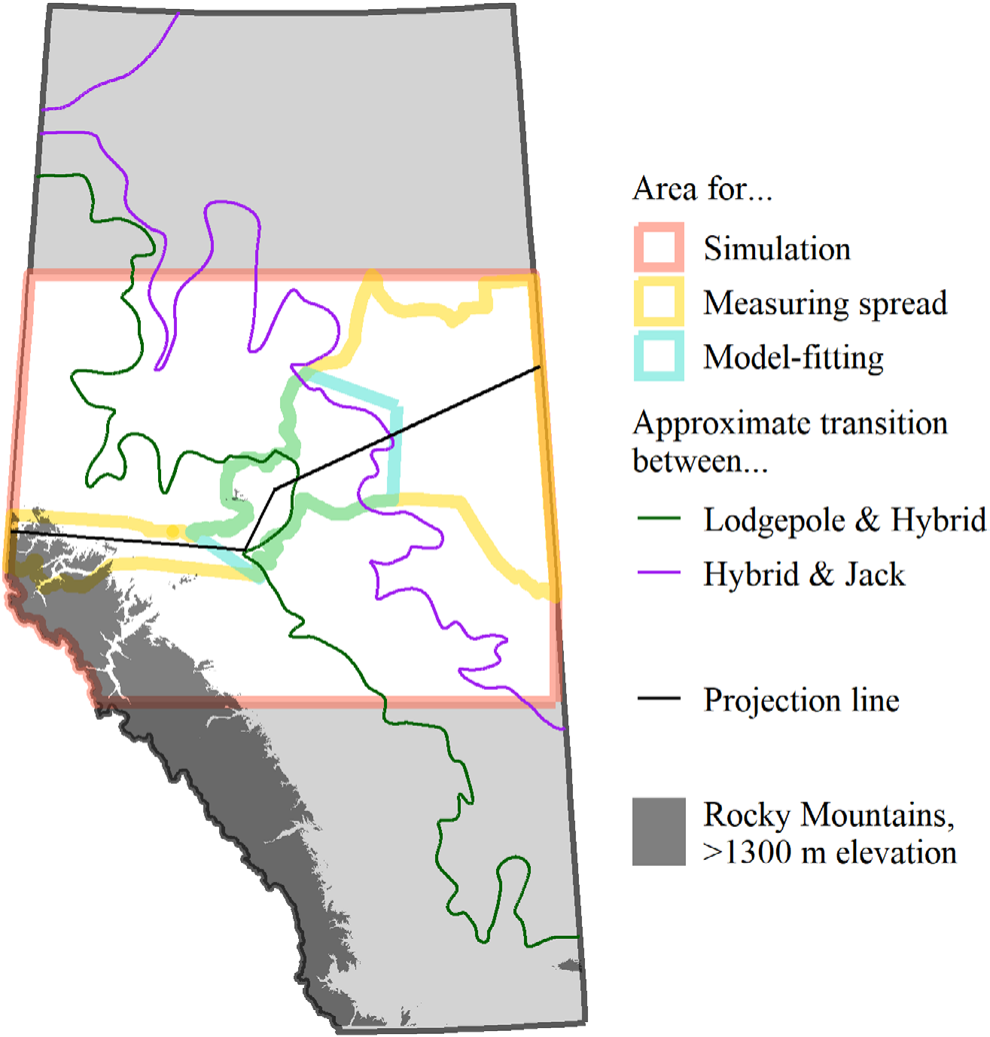
Different regions of Alberta are used for different parts of the analysis.

Using data from 2006 to 2015, the model forecasts infestations for 2007–2016. It does not attempt to predict 2006, because many of the 2006 infestations are thought to be caused by long‐distance dispersal from British Columbia (Carroll et al. 2017; Bartell 2008; Gayathri Samarasekera et al. 2012). Lastly, the study focused on a specific region of Alberta with approximately equal lodgepole‐dominant and hybrid/jack‐dominant pixels (Figure 5).

### 2.2 Model #1

We developed a statistical model to discern the differences between lodgepole pine and jack pine, with respect to their effective attack rate (i.e., how easy it is to find a suitable host, aggregate, and kill a tree) and effective brood size (i.e., average offspring per tree, multiplied by the average dispersal‐phase survival). The model aims to predict the number of trees infested with MPB, based on last year's infestations and the severity of the interceding winter.

First, we simulated MPB dispersal by convolving last year's infestations with a dispersal kernel. The result was *beetle pressure* $B_{t}\left(y\right)$, which is proportional to the expected number of beetles arriving at the destination pixel y:

(2) $$B_{t}\left(y\right)=\sum_{x}I_{t-1}^{*}\left(x\right)c\left(x\right)\theta_{t}\left(x\right)\bar{D}\left(\text{dist}\left(y,x\right)\right).$$

Here, $I_{t-1}^{*}\left(x\right)$ denotes the previous year's infestations that did not end up being sanitized; the parameter $c\left(x\right)$ denotes the effective brood size per infested tree, which varies with the species of pine present; and $\theta_{t}\left(x\right)$ denotes the probability of winter survival, pre‐computed using the model of Régnière and Bentz (2007). The quantity $I_{t-1}^{*}\left(x\right)\;c\left(x\right)\;\theta_{t}\left(x\right)$ is therefore proportional to the number of beetles produced in pixel x. The function $\bar{D}$ is the probability of dispersing from pixel x to pixel y. The sum over all pixels represents the discretization of a continuous‐space convolution using a midpoint Riemannian sum.

The underlying dispersal kernel is a Student's *t*‐distribution, which allows for a predominance of short‐distance dispersal and occasional long‐distance dispersal. The kernel density D is radially symmetric in 2D space, and therefore may be parameterized as a function of the Euclidean distance between two pixels, denoted $r=\text{dist}\left(x,y\right)$. The non‐marginal kernel density is given by

(3) $$D\left(r\right)=\frac{\left(\nu -1\right){\left(\frac{r^{2}}{{\nu \rho }^{2}}+1\right)}^{\frac{1}{2}\left(-\nu -1\right)}}{2{\pi \nu \rho }^{2}}.$$

Based on a previous study of MPB dispersal in western Alberta, the dispersal parameters were fixed at ν=1.45 and ρ=0.0118 (Johnson, Brush, and Lewis 2024). To accurately represent dispersal at a 1 km resolution, we convolve higher‐resolution grids (50 × 50 m pixels) of the discretized kernel density (i.e., $D\left(r\right)\times {\left(0.05\right)}^{2}$) and infestations that are uniformly distributed within the central square kilometer. The results of the convolution are aggregated to the 1 km scale, resulting in the transition probability function, $\bar{D}$ [see Equation (2)].

The effective brood size $c\left(x\right)$ varies based on the type of pine:

(4) $$c\left(x\right)=\left\{\begin{array}{cc} c_{L} & \text{if}\;x\in L,\\ c_{J} & \text{if}\;x\in J, \end{array}\right.$$

where L and J are the sets of all locations with lodgepole pine and jack/hybrid pine, respectively. The combination of two effective brood size parameters, along with all effective attack rate parameters (to be introduced shortly) is jointly unidentifiable. Therefore, we fixed the lodgepole pine brood size at $c_{L}=1$, and allowed $c_{J}$ to vary; this is precisely why we described $B_{t}\left(x\right)$ as *proportional* to the number of beetles. Only infested trees are observed, so the true number of beetles cannot be estimated from the survey data.

The “lodgepole pine pixels” L contained both lodgepole pine (Q>0.9) and hybrids (0.1≤Q<0.9). This grouping is the result of model selection—we tried grouping hybrid pixels with either jack or lodgepole pine pixels, and the latter resulted in better predictions according to leave one out cross validation (see Appendix S1.3). While the effective brood size could theoretically be a continuous function of Q, this would require computationally expensive convolution at each iteration of the model‐fitting procedure. Discretization allowed us to use fast Fourier transforms for dispersal calculations just once before model fitting begins. Then, we simply multiplied the J dispersal surface by $c_{J}$.

To predict next year's infestations from beetle pressure, we adopted a *zero‐inflated negative binomial* model. This choice naturally accommodates two key features of the data: (1) a high proportion of sites with no infestations (i.e., zero‐inflation), and (2) overdispersion in the count data (i.e., the variance exceeds the mean number of infestations). The high proportion of zeros likely reflects high levels of dispersal mortality (Safranyik 1989; Latty 2007; Pope et al. 1980) and within‐pixel aggregation. Zero‐inflated negative binomial models are common in ecology and have previously been used to model MPB (Xie 2024).

The partial probability of observing an infestation, denoted $\pi_{t}\left(x\right)$, was modeled using logistic regression. This presence/absence sub‐model only generates the *potential* for a non‐zero number of infestations, as the count model may still generate zeros.

(5) $$\begin{array}{c} \pi_{t}\left(x\right)=\left\{\begin{array}{cc} {\text{logit}}^{-1}\left(\gamma_{0,L}+\gamma_{1,L}\log\left(B_{t}\left(x\right)\right)\right) & \text{if}\;x\in L,\\ {\text{logit}}^{-1}\left(\gamma_{0,J}+\gamma_{1,J}\log\left(B_{t}\left(x\right)\right)\right) & \text{if}\;x\in J. \end{array}\right. \end{array}$$

The number of infestations follows a negative binomial distribution, parameterized by the mean (μ) and the dispersion parameter (ϕ):

(6) $$\begin{array}{c} f\left(I_{t}\left(x\right)\right)=\left\{\begin{array}{cc} \mathrm{NB}\left(I_{t}\left(x\right)\;|\;\mu =\exp\left[\beta_{0,L}+\beta_{1,L}\log\left(B_{t}\left(x\right)\right)\right],\phi =k_{L}\right) & \text{if}\;x\in L,\\ \mathrm{NB}\left(I_{t}\left(x\right)\;|\;\mu =\exp\left[\beta_{0,J}+\beta_{1,J}\log\left(B_{t}\left(x\right)\right)\right],\phi =k_{J}\right) & \text{if}\;x\in J. \end{array}\right. \end{array}$$

Here, $\mathrm{NB}\left(y\;|\;\mu ,\phi \right)$ denotes the probability mass function:

(7) $$\mathrm{NB}\left(y\;|\;\mu ,\phi \right)=\left(\begin{array}{c} y+\phi -1\\ y \end{array}\right){\left(\frac{\mu }{\mu +\phi }\right)}^{y}{\left(\frac{\phi }{\mu +\phi }\right)}^{\phi }.$$

The overall probability of observing a number of infestations is a mixture of the zero‐inflated and the negative binomial components:

(8) $$\begin{array}{c} \mathrm{Pr}\left(I_{t}\left(x\right)\right)=\left\{\begin{array}{cc} \left(1-\pi_{t}\left(x\right)\right)+\pi_{t}\left(x\right)\cdot f\left(I_{t}\left(x\right)\right) & \text{if}\;I_{t}\left(x\right)=0,\\ \pi_{t}\left(x\right)\cdot f\left(I_{t}\left(x\right)\right) & \text{if}\;I_{t}\left(x\right)>0. \end{array}\right. \end{array}$$

The structure of the statistical model is justified by graphical evidence and prior knowledge. The logistic relationship in the presence/absence model and the log–log linear relationship in the count model emerge from exploratory plots (Figure S2.1), and the negative binomial distribution accurately accounts for residual error (Figure S2.2). The model structure is also biologically sensible. Mountain pine beetle experiences positive density dependence in the form of an Allee effect, in that a threshold number of beetle attacks are required to overcome tree defenses (Thalenhorst 1958; Raffa and Berryman 1983; Boone et al. 2011). Mountain pine beetle also experiences negative density dependence at larger spatial scales, putatively due to the difficulty of finding suitable host trees that are not already filled up with beetles (Trzcinski and Reid 2009; MacQuarrie and Cooke 2011). Both kinds of density dependence occur at the host‐finding/selection and mass attack stages of MPB's life cycle, and are therefore plausibly captured by the *effective attack rate parameters*, for example, $\beta_{0,L},\beta_{1,L},\gamma_{0,L},$ and $\gamma_{1,L}$ for lodgepole pine alone.

The *effective brood size* is represented by a single parameter (e.g., $c_{L}$ for lodgepole pine) because it appears to be independent of beetle pressure, as shown in the *Alberta disk data* (Appendix S3). This consistency in brood size is likely due to the mountain pine beetle (MPB) maintaining a near‐optimal attack density on successfully attacked trees (approximately 60 attacks per square meter; Raffa and Berryman 1983), regardless of overall beetle population pressure. Beetles achieve this density through two main mechanisms: anti‐aggregation pheromones, which direct excess beetles to nearby trees, and male stridulations (sound signals), which help space galleries evenly (Safranyik and Carroll 2006). Beetles that ignore these signals have lower fitness, possibly due to interference competition with established mating pairs and the poor quality (i.e., thin phloem) of the remaining uncolonized areas (Raffa 2001).

### 2.3 Model #2

We created a second model to determine whether MPB is more influenced by pine species identity or structural differences between the forests of western and eastern Alberta. Pine ancestry (*Q*‐values) captured differences between the two pine species with respect to species‐level characteristics like tree size, phloem thickness, and monoterpene concentrations. Pine volume served as a proxy for forest structure, capturing the west‐to‐east gradients in tree size, tree density, forest composition, and pine connectivity. The model generally has the same structure as Model #1—a dispersal step to calculate beetle pressure, followed by a zero‐inflated negative binomial distribution—with a few notable differences. First, we fixed both brood size parameters at $c_{L}=c_{J}=1$, which was based on the fact that $c_{J}$ could not be estimated precisely by Model #1, and the fact that prior bolt experiments indicate similar brood sizes (see the *Results*). Second, instead of allowing for pine‐specific attack rate parameters (e.g., $\gamma_{J}$ & $\gamma_{L}$), we use both pine ancestry and pine volumes as predictor variables.

The probability of potentially observing a non‐zero number of infestations [previously Equation (5)] now becomes

(9) $$\text{logit}\left(\pi \right)=\gamma_{0}+\gamma_{B}B^{*}+\gamma_{Q}Q^{*}+\gamma_{V}V+\gamma_{\mathrm{QV}}\mathrm{QV},$$

where $B^{*}$ $Q^{*}$ and V are respectively the beetle pressure, pine ancestry, and pine volume predictors. Dropping the explicit notation for time and location, and defining the standardization operator $\mathrm{std}\left(\left(x-\bar{x}\right)/\mathrm{sd}\left(x\right)\right)$, we can write the predictors as $B^{*}=\mathrm{std}\left(\log\left(B\right)\right)$, $Q^{*}=\mathrm{std}\left(Q\right)$, $V=\mathrm{std}\left(\log\left(\text{pine}\;\mathrm{vol}\right)\right)$. The log‐transformations were necessary to meet linearity assumptions, and standardization allowed us to interpret the magnitude of the coefficients as predictor importance.

A similar modification was made to the count model [previously Equation (6)]; the mean of the negative binomial distribution becomes

(10) $$\mu =\exp\left[\beta_{0}+\beta_{B}B^{*}+\beta_{Q}Q^{*}+\beta_{V}V+\beta_{\mathrm{QV}}\mathrm{QV}\right].$$

Again, the structure of model #2 is justified by graphical evidence: the predictors $B^{*}$, $Q^{*}$, and V all have approximately linear relationships with $\text{logit}\left(\pi \right)$ and $\log\left(\mu \right)$ (Figure S2.3). The types of model inputs used across both model #1 and model #2—beetle densities, winter temperatures, pine species, and pine volume/density—are consistently found as the most important predictors of MPB dynamics (Aukema et al. 2008; Ramazi et al. 2021; Srivastava and Carroll 2023). As a robustness check, a variant of model #2 with interaction effects is analyzed in Appendix S1.4.

Both model #1 and model #2 were parameterized using *Stan*, a Bayesian model‐fitting program. Standard model‐fitting diagnostics were examined. Additionally, the posterior contraction statistic demonstrated that prior distributions generally had a small influence on parameter estimates (Appendix S1.5).

### 2.4 Simulations

We simulated both models across the entire longitudinal extent of Alberta (Figure 5), using the 2006 infestations as the initial state (Figure S2.10). Infestations were not allowed in pixels where pine volume was less than 1 m^3^km^−2^. To simulate the provincial control efforts, we define $m_{t}$, the proportion of infestations that were controlled in year t (within the polygon for measuring spread; Figure 5). Then, the calculation of beetle pressure [previously Equation (2)] becomes

(11) $$B_{t}\left(y\right)=\sum_{x}I_{t-1}\left(x\right)m_{t-1}c\left(x\right)\theta_{t}\left(x\right)\bar{D}\left(\text{dist}\left(y,x\right)\right),$$

where $I_{t-1}\left(x\right)\times m_{t-1}$ is the simulated number of uncontrolled infestations.

To measure MPB spread, we took all data within the *Area for measuring spread* (Figure 5) and projected it onto a *projection line* that runs west‐to‐east across Alberta. Then, eastward spread was measured as the 99th percentile of eastward distance along this line. The *Area for measuring spread* polygon is the intersection of surveyed areas across years, and thus allows for a fair comparison between real and simulated data; the projection line was intentionally drawn to traverse through the middle of this polygon.

The simulations served several purposes. First, a comparison of actual data and simulated data helped validate the models. Second, simulations quantified the uncertainty in the eastward spread of MPB infestations. Each simulation was instantiated with a random sample from the joint posterior distribution of model parameters, and thus variation across simulations represents both parameter uncertainty and accumulated process error. Third, we performed a variety of such counterfactual simulations for both model #1 and model #2, respectively focused on teasing out the relative importance of the effective attack rate versus the effective brood size, and pine ancestry versus pine volume.

The statistical models did not account for the depletion of suitable pine hosts, but we included this factor in the simulations. We imposed a limit on the number of trees that can be infested in a single pixel, as otherwise, an exponential buildup in some locations could create unrealistic beetle pressure. The maximum number of trees that can be infested per pixel, across all years, is set to the 99th percentile of cumulative infestations across all pixels containing lodgepole pine (i.e., Q>0.9) and at least one infestation. Resource depletion is not included in the statistical model for pragmatic reasons, and because it is unclear whether the effects of resource depletion are estimable—there is no apparent negative relationship between next year's infestations and the cumulative number of trees killed by beetles (Figure S2.5). MPB populations have declined across Alberta, but this may be primarily attributable to the provincial government's control efforts or several unusually cold winters from 2018 to 2023, rather than host tree depletion via beetle attack.

## 3 Results

Our results indicate that MPB's slow spread through jack pine is attributable to a low effective attack rate, not a low effective brood size. The first statistical model was not able to accurately estimate the effective brood size, but experimental studies strongly suggest that the two pine species have similar effective brood sizes (Safranyik and Linton 1982; Cerezke 1995; Rosenberger et al. 2017; Musso 2023). The second statistical model implies that the low effective attack rate in jack pine is due to jack pine's characteristics *and* the lower pine volumes of eastern Alberta; however, pine species identity is the more important predictor.

The effective brood size cannot be discerned using Model #1. Specifically, the effective brood size in jack pine is estimated with wide credible intervals, predicting 30%–160% of the effective brood size in lodgepole pine (Table S2.1). The reason for this large uncertainty is simple: there are so many more infestations in lodgepole pine that the total beetle pressure is dominated by contributions from lodgepole pine stands, regardless of the jack pine's brood size multiplier (i.e., the parameter $c_{J}$).

To probe the robustness of this result—the ineffectiveness of models in estimating the effective brood size—we examined the posterior distribution of $c_{J}$ across a number of different subjective modeling decisions (Appendix S1.3). While some models expressed confidence (i.e., narrow posteriors), they disagreed on whether jack pine or lodgepole pine had the larger effective brood size. This disagreement further demonstrates that models are not effective for estimating $c_{J}$.

**FIGURE 6:**
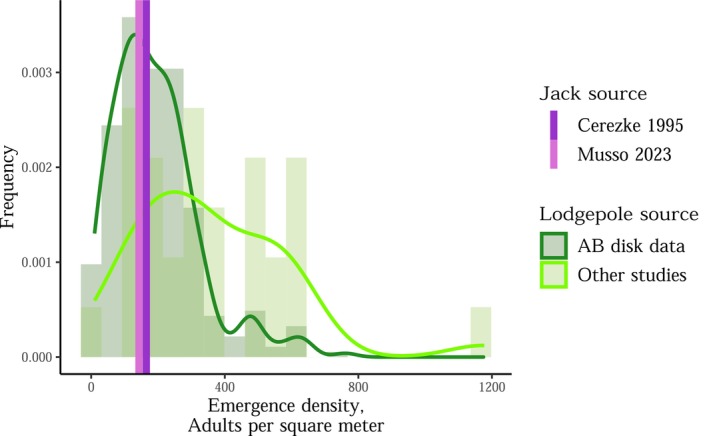
Jack pine and lodgepole pine show similar emergence densities, though jack pine data are limited. The two studies assessing emergence density in jack pine (Cerezke (1995) and Musso (2023)) are bolt experiments where bolts or live trees (which were later cut into bolts) were mass attacked by MPB in situ. The AB disk data contain emergence densities estimated from 2 in. diameter disks of bark from infested lodgepole pine across Alberta (Government of Alberta 2016), and “Other studies” contain field measurements (entry holes, emergence holes, emergence into cages) from naturally infested trees: Tishmack et al. (2005); Negrón (2018); Reid (1963); Raffa and Berryman (1983); Rasmussen (1980); De Leon (1939); Safranyik (1988); W. E. Cole (1969); Schmid (1972); Whiteside (1937); Beal (1938); Peterman (1974); Klein et al. (1978); Safranyik and Linton (1985, 1991).

Bolt experiments show that the effective brood size is similar in lodgepole pine and jack pine (Table 1). Of the five experiments that have been conducted in both pine species, four studies find similar MPB fitness. The exceptional study (Bleiker et al. 2023) only used a single bolt per pine species, so the result may be due to a tree or bolt effect, rather than a species effect. Of the two experiments that measured emergence density (progeny per tree surface area), both found similar emergence densities in lodgepole pine and jack pine (Cerezke 1995; Bleiker et al. 2023). Further, experimental emergence densities in jack pine bolts are similar to the median emergence density in lodgepole pine in Alberta, per the *Alberta disk data* (Figure 6). Attack densities in jack pine and lodgepole pine show a similar pattern (Figure S2.6), further supporting the idea that MPB treats both pine species similarly once mass attacks are underway. Finally, beetle body size and sex ratios are generally similar in lodgepole and jack pine (Table 1).

**TABLE 1:** The effective brood size of MPB is similar in lodgepole pine and jack pine: Evidence from bolt experiments.

Data source	Pine species	Fitness proxy, brood/female	Emergence density, adults per m^2^	Pronotum width, female/male, in mm	Proportion female	No. mating pairs introduced	No. trees	No. bolts	Phloem thickness, mm	DBH, cm	Successful galleries
Safranyik and Linton (1982)	Lodgepole	31.6		2.12/1.89^a^	0.64						
	Jack	36.5		2.09 (0.05)/1.90 (0.032)^a^	0.81	20	1	4		19.9	65%
Cerezke (1995)	Lodgepole	_3.94_ ^b^	129	2.13/1.91	0.625		2	6		19–23^c^	
	Jack	_2.59_ ^b^	165	1.99/1.79	0.513		2	6		19–23^c^	
Rosenberger et al. (2017)	Lodgepole	22.9 (3.2)^d^			0.553		8	8		23–30^c^	
	Jack	27.8 (4.2)^d^			0.549		8	8		23–30^c^	
Bleiker et al. (2023)	Lodgepole	38.4 (6.1)		1.97 (0.02)/1.76 (0.02)	0.5	26	1	1	1.3	30–40^c^	54%
	Jack	15.1 (5.0)		2.01 (0.01)/1.82 (0.02)	0.625	35	1	1	1.6	30–40^c^	49%
Musso (2023), ch. 2 Musso (2023), ch. 3	Lodgepole	2.35 (0.976)^b^	139 (5.1)	1.99 (0.086)	0.673	14	28	13	1.99 (0.086)	26.2 (0.48)	
	Jack	3.82 (0.558)^b^	143 (36.8)	1.4 (0.056)	0.588	18	36	13	1.4 (0.056)	29.0 (0.54)	

*Note:* Values are given as mean ± standard error (if available). Blank cells represent N/A values. Only two studies (Cerezke 1995; Musso 2023) attempted to simulate mass attacks; these studies resulted in lower fitness measurements, putatively due to higher intraspecific competition between larvae. The remaining studies inoculated mating pairs into pre‐drilled holes. One study was considered but excluded due to the presence of saprophytic fungi in some of the bolts used (Lusebrink et al. 2016).

^a^ Prothorax width, not pronotum width.

^b^ Emerging adults per female, not under‐bark brood per female.

^c^ DBH range is across lodgepole and jack pine bolts together.

^d^ Midwinter (January) brood as opposed to spring/summer pre‐flight brood.

**FIGURE 7:**
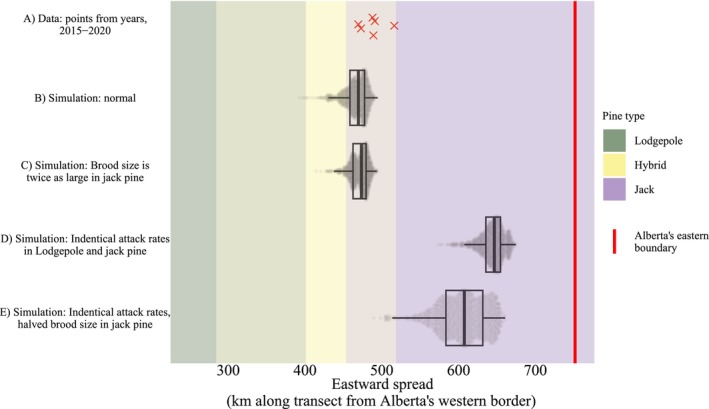
Mountain pine beetle's slow spread is due to a low attack rate in jack pine: Evidence from counterfactual simulations of model #1. The *y*‐axis shows the data and different simulation scenarios. The *x*‐axis shows the eastward spread of MPB as measured by the 99th percentile of distances for infestations, from 2015 to 2020, projected along the projection line (see Figure 5). Overlap in pine type colors represents transition zones between pine types that result from projecting curved 2D species boundaries onto 1D transect line. For computational details, see the Files S1.

Counterfactual simulations of Model #1 imply that a low effective attack rate is responsible for MPB's slow spread through jack pine. When we change the attack rate parameters in jack pine so that they are equal to the corresponding attack rate parameter in lodgepole pine, MPB moves quickly through eastern Alberta (Figure 7, row E). In contrast, increasing the effective brood size to 2 × larger in jack pine (compared to lodgepole pine) does not substantially increase the spread distance (Figure 7, row C). This indicates that MPB has such a low attack rate in jack pine forests, that even a large brood size cannot compensate for it. If the effective brood size is 2 × *smaller* in jack pine, but MPB has identical attack rates in both pine species, then MPB still spreads through Alberta (Figure 7, row D). Rows C and D respectively show that having similar effective brood sizes in lodgepole and jack pine is neither sufficient nor necessary for the spatial spread of MPB through jack pine.

**FIGURE 8:**
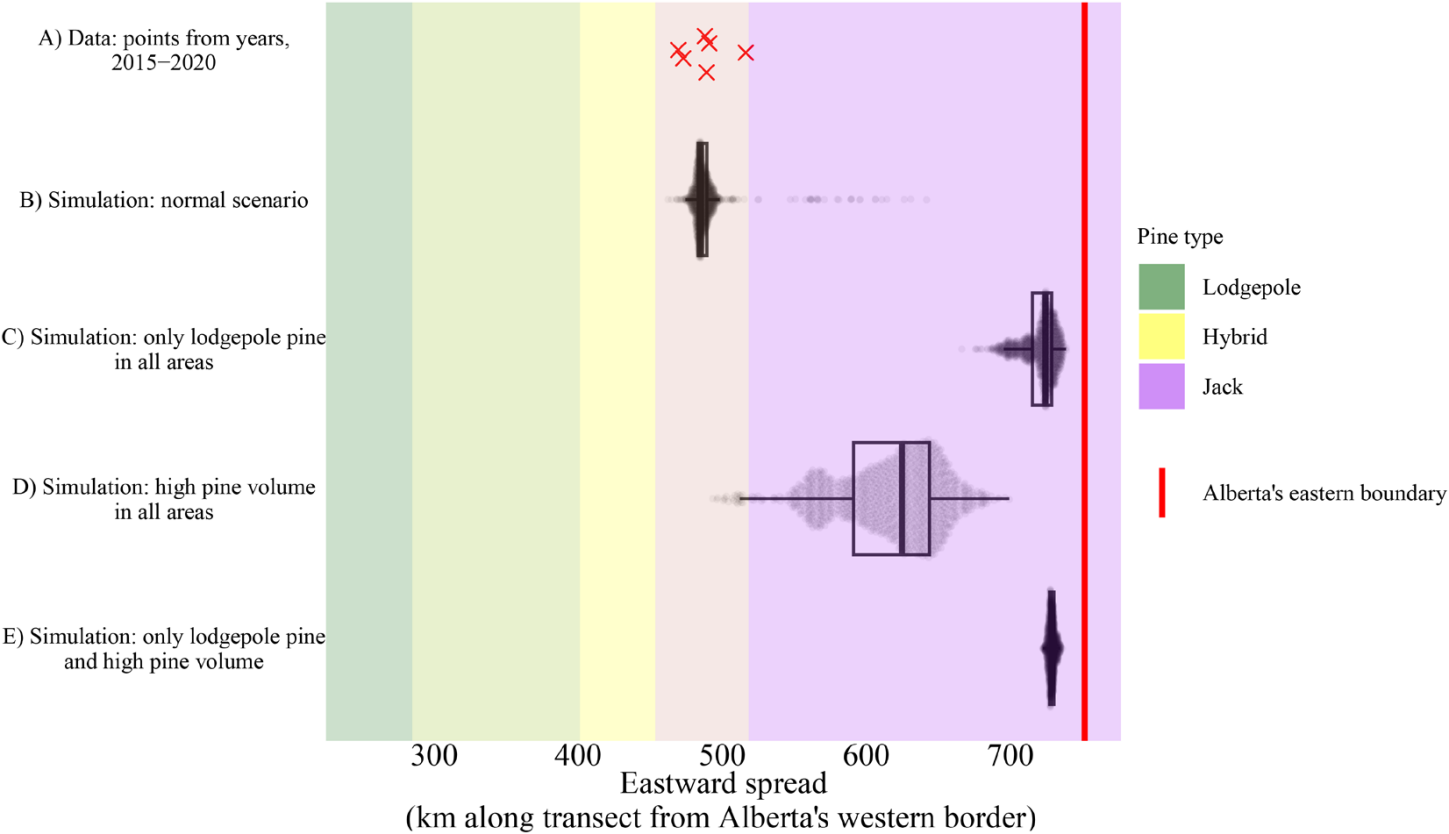
Mountain pine beetle's slow spread is primarily due to some species‐level property of jack pine: Evidence from counterfactual simulations model #2. The *y*‐axis shows the data and different simulation scenarios. The *x*‐axis shows eastward spread of MPB as measured by the 99th percentile of distances for infestations, from 2015 to 2020, projected along the projection line (see Figure 5). Overlap in pine type colors represents transition zones between pine types that result from projecting curved 2D species boundaries onto 1D transect line. For computational details, see the Files S1.

Counterfactual simulations of Model #2 imply that jack pine is responsible for MPB's slow spread through eastern Alberta. When we model a scenario where pine is everywhere—setting the pine ancestry predictor to Q=1 throughout the simulation area—MPB spreads further eastward in comparison to the baseline simulations (Figure 8, row C). To investigate the effect of low pine volumes in eastern Alberta, we imagine that pine volume in western Alberta is replicated everywhere—all pixels with pine (i.e., pine volume $>1m^{3}{\mathrm{km}}^{-2}$) are assigned the mean pine volume of lodgepole pine forests (Figure 8, row D); the mean is calculated across pixels within the area for measuring spread (see Figure 5) where Q>0.9, and pine volume $>1m^{3}{\mathrm{km}}^{-2}$. Here, we see a spread distance that is greater than the normal scenario, but less than the “lodgepole everywhere” scenario. This suggests that both pine species and pine volume matter, but that the pine species identity matters more.

## 4 Discussion

The slow spread of mountain pine beetle (MPB) through eastern Alberta is due to an inability to find and attack jack pine, which in turn is largely caused by some species‐level properties of jack pine. Importantly, our results contrast with a commonly accepted view, wherein the slow spread of MPB is attributed to low effective brood sizes (i.e., poor reproduction and development) and lower pine volumes. For instance, a recent national (Canadian) risk assessment concluded that “The slower rate of spread is attributed to lower pine volumes, poor connectivity of susceptible pine stands, lower probability of long‐distance dispersal events as distance to a large source population increases, and aggressive control efforts sustained to date by the province in eastern Alberta” (Bleiker et al. 2023). An earlier paper had a similar conclusion: “Jack pine stands in the boreal zone are less susceptible to outbreaks than are lodgepole pine stands in western Canada because of relatively lower pine volume in these stands.” (Safranyik et al. 2010). In contrast, we find that landscape‐scale differences in pine volume play a secondary role in the eastward spread of MPB.

A variety of mechanisms can be invoked to explain MPB's differential response to lodgepole versus jack pine. In the following paragraphs, we discuss mechanisms as they apply to different stages of the MPB life cycle. An executive summary with subjective certainty ratings is provided in the *Conclusions*.

### 4.1 MPB Development and Reproduction

Previous studies have identified several mechanisms affecting MPB development in jack and lodgepole pine, including differences in phloem thickness and nitrogen content. Jack pine tends to have thinner phloem than lodgepole pine due to its smaller diameters and lower phloem thickness at the same diameters (D. M. Cole 1973; Lusebrink et al. 2016; Figure S2.7). Since MPB larvae consume phloem as their primary source of nutrition, thin phloem could plausibly lead to slower development, more overwintering mortality, smaller body sizes, and less fat content (D. M. Cole 1973; Musso et al. 2023). Jack pine phloem has 30% lower nitrogen content, potentially impacting larval growth and development (Lusebrink et al. 2016).

While thinner phloem and lower nitrogen content in jack pine imply slower MPB development and higher overwintering mortality in theory, these factors seem to have a minor impact in reality, putatively because of compensatory behaviors observed in MPB larvae. Musso (2023; Ch. 3) found that larvae in jack pine compensate for thinner phloem by consuming across a greater area of the bole; the total volume of phloem consumed is actually greater in jack pine than in lodgepole pine. Jack pine is poorly defended, with lower levels of constitutive defensive compounds compared to lodgepole pine (Clark et al. 2014), but this does not seem to have a net effect on MPB fitness. Perhaps reduced mortality from resinosis is counteracted by intraspecific competition between larvae (Raffa and Berryman 1983), or brood mortality from resinosis is simply not a large component of total mortality (Amman 1983).

Jack pine also produces lower levels of *induced* defensive compounds in response to MPB attacks (Musso et al. 2023) and inoculation with MPB's symbiotic blue‐stain fungus (Arango‐Velez et al. 2016; Lusebrink et al. 2016; Erbilgin et al. 2017), which should reduce larval mortality due to toxicity. This mechanism is supported by evidence from lodgepole pine: trees in the historic range exhibit increased terpene concentrations six weeks post‐attack (Clark et al. 2012), while trees in the expanded range show no such increase (Musso et al. 2023), and this difference in induced responses may explain higher MPB fitness in naïve populations (Cudmore et al. 2010). By extension, jack pine's comparatively weak induced defenses may enhance MPB fitness. Cut bolts do not exhibit induced defenses in either species of pine tree, potentially obscuring higher MPB survival in jack pine. However, the total extent and direction of experimental biases introduced by cut bolts remain uncertain.

Some bolt experiments in Table 1 attempt to control for tree size by selecting lodgepole and jack pine bolts within a narrow DBH range. In reality, jack pine trees typically have a 2.5–5 cm smaller DBH than lodgepole pines, on average (Nunifu 2009, table 1; Strimbu et al. 2017, table 1; Burns 1990, 617 & 566). The smaller average size of jack pine could result in a slightly reduced effective brood size compared to lodgepole pine, but given the small difference in average sizes, and the fact that MPB seems to have positive fitness on smaller (i.e., 20–30 cm DBH) lodgepole pines (Johnson, Musso, et al. 2024), this possible difference is likely not large. Additionally, the aforementioned effect of jack pine's lower induced defenses, which is similarly not reflected in bolt experiments, may counteract any size‐related effects.

Another limitation of bolt experiments is that storage and handling protocols may introduce biases or variation between studies that can be hard to interpret. Most bolt experiments allow beetle progeny to overwinter at 5°C, a temperature that is considerably warmer than natural conditions. While this practice isolates treatment effects and minimizes background mortality, it may mask important tree species × cold stress interactions. For example, lodgepole pine's thicker bark may provide inadequate insulation for overwintering beetles under natural conditions, leading to higher cold‐induced mortality that would not manifest in laboratory settings. However, Rosenberger et al. (2017) found similar MPB survival in lodgepole and jack pine bolts exposed to natural temperature variation. An opposing effect of “warm overwintering” is that 5°C slows beetle development while still permitting saprophytic fungi to grow, which could degrade MPB habitat. Although speculative, this mechanism could explain the counterintuitive observation that MPB fitness is higher in bolts exposed to natural winter temperatures (compare Musso 2023, ch. 5 and Rosenberger et al. 2018).

### 4.2 Host Finding/Selection

Due to its chemical profile, jack pine may be harder to locate during the host‐finding/selection stage. Jack pine has lower relative and total levels of *β*‐phellandrene (Miller and Borden 1990; Clark et al. 2014; Burke and Carroll 2016), the only monoterpene known to play a role in primary attraction (Miller and Borden 1990). However, it is unknown how important olfaction is to MPB during the host‐finding/selection stage compared to other senses (Reid 1963; Raffa and Berryman 1982; Raffa 2001). While lower *β*‐phellandrene levels might reduce jack pine's attractiveness to MPB, high beetle pressures likely mitigate this effect, since only a few attacking females are needed to initiate a mass attack.

It is well‐established that MPB respond differentially to various types and concentrations of terpenes and pheromones (Borden et al. 1983, 1987; Conn et al. 1983; Pureswaran and Borden 2005; Campbell and Borden 2006), but comparatively little is known about host‐seeking behavior with a choice between lodgepole pine and jack pine. The most direct evidence to date comes from choice experiments where beetles were presented with either air streams containing volatiles from jack pine or lodgepole pine phloem tissue, or whole bolts of both species in arena trials (Musso 2023, ch. 5). Beetles showed no apparent preference between the two host species in either experimental setup.

### 4.3 Mass Attacks

During MPB mass attacks, differences in the chemical profiles of lodgepole and jack pine have been hypothesized to influence MPB behavior. Jack pine exhibits higher relative concentrations of *α*‐pinene, the precursor to MPB's primary aggregation pheromone, *trans*‐verbenol (Hughes 1973; Chiu et al. 2018), and female MPB colonizing jack pine in the lab produce 2 × more *trans*‐verbenol (Erbilgin et al. 2014). Jack pine also has lower relative concentrations of myrcene and higher levels of 3‐carene, terpenoid volatiles that act synergistically with aggregation pheromones to attract beetles (Erbilgin et al. 2014; Seybold et al. 2006; Chiu and Bohlmann 2022). Among these interspecific differences, the most biologically significant is thought to be *α*‐pinene, due to its status as a precursor to aggregation pheromones, and due to the fact that its relative concentration is 3–4 times higher in jack pine than in lodgepole pine (Burke and Carroll 2016; Erbilgin et al. 2014).

Despite an abundance of *α*‐pinene in jack pine, we speculate that jack pine is still difficult to mass attack due to lower total concentration of other crucial monoterpenes. While there is some experimental evidence that MPB behavior is more influenced by the relative concentration/composition of monoterpenes (reviewed in Erbilgin 2019), the reliance on bolts or pheromone traps in these studies raises questions about the generalizability of the findings to the real world. In contrast, lodgepole trees that are naturally attacked during an MPB epidemic have nearly twice the total concentration of monoterpenes, in comparison with non‐attacked trees (Boone et al. 2011). Across various similar studies involving different bark beetle species and their host trees, total monoterpenes are consistently better predictors of beetle attack than specific monoterpene composition (8 out of 15 vs. 2 out of 15 studies; see Howe et al. 2024, table 4). It is likely that total monoterpene concentration, which is typically measured in units of mg/g phloem in the immediate vicinity of boring holes, is a proxy for the chemical environment on a larger spatial scale.

We propose that the physical characteristics of jack pine lead to smaller plumes of attractive volatiles around mass‐attacked trees, thus leading to a low effective attack rate. Together, jack pine's smaller size and lower total monoterpenes may result in smaller plumes of attractive volatiles around mass‐attacked trees, making it more challenging for MPB to successfully execute mass attacks. It is difficult to say how much of jack pine's smaller size and thinner phloem is a species‐level trait due to the confounding effect of the environment; jack pine tends to grow at lower elevations with well‐drained soil and southern exposure, leading to nutrient‐poor soil (Cullingham et al. 2012; Burns 1990).

Supporting evidence for this mechanism—tree‐size‐dependent pheromone/volatile plumes—comes from extensive studies on lodgepole pine. Invariably, MPB outbreaks end before most medium‐sized lodgepole pines are killed, despite beetles having positive fitness when attacking medium‐sized trees (Johnson, Musso, et al. 2024, fig. 5 and 6); this implies that MPB needs a strong chemical signal (i.e., such as those provided by large trees) to successfully aggregate. Tree diameter is a clear predictor of tree mortality (reviewed by Björklund and Lindgren 2009). Stand‐level maps of infestations reveal that beetles only attack medium‐sized trees when they are adjacent to large trees (Mitchell and Preisler 1991; Preisler and Mitchell 1993). Mechanistic models of pheromone diffusion imply that pheromones can be detected 20–50 m from their source tree, supporting the general idea that MPB has a limited ability to detect host trees (Biesinger et al. 2000; Strohm et al. 2013). Further evidence comes from a network of pheromone‐baited trees in eastern Alberta and western Saskatchewan. Small numbers of beetles were found near the Alberta‐Saskatchewan border in 2017 (Bleiker 2019), much farther east than any mass‐attacked tree, suggesting that aggregating on host trees is a limiting factor.

Our results show that pine volume is not the best predictor of MPB spread, which speciously contradicts the idea that the lower attack rate on jack pine is partly due to jack pine's smaller size; after all, pine volume can be mathematically represented as the average volume per pine, multiplied by the total number of pines. However, the correlation between pine volume and pine crown cover was r=0.75, indicating that pine volume mostly tracks the density and distribution of trees, not tree size. It is unclear whether pine volume across a ${\mathrm{km}}^{2}$ pixel is a relevant metric—from the perspective of MPB, what matters is the availability of dense stands of large‐diameter pines. Future studies could attempt to distinguish between the effects of tree size and other species‐specific properties (like thinner phloem and lower monoterpene concentrations in jack pine) using stand‐level data of tree diameter distributions.

### 4.4 Alternative Hypotheses for Slowed Spread

There are several alternative explanations for the slow spread of MPB in eastern Alberta, but we do not find evidence for these explanations in our data. One explanation is that the climate of the jack pine forest is unsuitable for MPB. While northern boreal forests are too cold for MPB survival (Carroll et al. 2004; Cooke and Carroll 2017), mid‐latitude Alberta jack pine forests have a suitable climate, comparable to lodgepole pine in the Rocky Mountain foothills. This is partially because the lower elevation of eastern Alberta compensates for the higher latitudes where jack pine is found (Figure S2.8).

Another explanation attributes the slowed spread to the provincial government's control strategy of cutting and burning infested trees. However, despite control efforts starting in 2006, MPB continued rapid spread until 2010. Moreover, the highest proportion of controlled infested trees was in western Alberta (Figure S2.9). While management actions may have limited total tree mortality (Carroll et al. 2017) and prevented some eastward spread, they were probably not the primary factor in the significant slowdown around 2009.

A third explanation attributes the rapid eastward expansion to long‐distance dispersal from BC, with the slowing spread in Alberta reflecting BC's declining hyperepidemic. A population genetic analysis found that beetles in 2006 and 2007 had different origins compared to those in 2005 (Gayathri Samarasekera et al. 2012), suggesting that the 2006 expansion is at least partially due to long‐distance dispersal. However, it seems unlikely that subsequent expansions (such as those in 2009 or 2011) were the consequence of long‐distance dispersal from British Columbia, given the 500 km distance between central BC and central Alberta, as well as decreasing infestation densities in BC.

## 5 Conclusions

**FIGURE 9:**
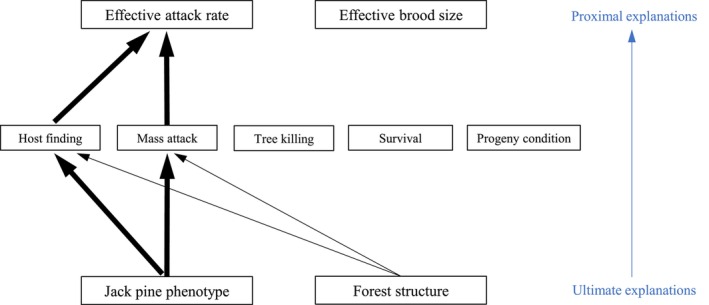
Conclusions, visualized as a simplification of Figure 4. Some species‐level property of jack pine (putatively its smaller size, thinner phloem, and lower total monoterpene concentrations) negatively affects MPB's ability to find and/or mass attack jack pine trees. The thicker arrows indicate a putatively larger effect. The mediating arrow through “Tree killing” has been removed because it is assumed that there are always sufficient beetles to overcome tree defenses during the epidemic phase of an outbreak. All arrows indicate positive effects.

Our main findings and their interpretations with subjective confidence ratings (low, moderate, and high) are depicted in Figure 9 and expounded below:

1. MPB's range expansion has slowed because MPB has difficulty in finding and/or mass‐attacking jack pine in the forests of eastern Alberta, not because host trees are inhospitable in terms of beetle reproduction and development; in the jargon of this paper, MPB has a lower *effective attack rate* in jack pine forests than in lodgepole pine forest (high confidence); but MPB does not have a lower *effective brood size* in jack pine forests (moderate confidence).
2. MPB has similar fitness and brood size in jack pine and lodgepole pine (moderate confidence). Bolt experiments show similar emergence densities and per capita emergence rates across 4/5 studies, with the exceptional study only using a single bolt per pine species. However, confidence in this conclusion is tempered by the modest number of studies and the limitations of cut bolt experiments: beetles may not interact with cut bolts the same way they interact with live trees, cut bolts lack induced defenses, and cut bolts are often stored at unrealistically warm temperatures. Additionally, there are opposing biases that may mask species‐level differences. Jack pine has thinner phloem than lodgepole pine, which ought to negatively impact MPB development and survival. However, the majority of bolt experiments (4 out of 5) did not attempt to control for phloem thickness, but still found similar brood sizes in lodgepole and jack pine. It is possible that MPB larvae compensate by simply eating more phloem (low confidence: only a single study measured phloem consumption). It is also possible that the deleterious effects of jack pine's thinner phloem are counteracted by lower levels of constitutive and induced defenses (i.e., toxic monoterpenes) in jack pine (low confidence, bolt experiments cannot assess induced defenses).
3. Pine species identity was a better predictor of MPB spread than pine volume. This is possibly due to the semiochemical profile of jack pine—specifically lower relative and total concentrations of *β*‐phellandrene—making host‐finding more difficult (low confidence). The slightly more plausible mechanism, however, involves a combination of jack pine's smaller size, thinner phloem, and lower total monoterpene concentrations; these traits result in smaller plumes of attractive volatiles during the mass attack stage, thus decreasing the probability of a successful attack (moderate confidence).

The notion that jack pine is inherently unsuitable for mountain pine beetle (MPB) should be approached cautiously. The most probable scenario for further range expansion is a long‐distance dispersal event from high‐volume lodgepole pine stands in central Alberta to high‐volume jack pine stands in western Saskatchewan (Bleiker 2019; Hodge et al. 2017). Our results might be misinterpreted to suggest that MPB management in central Alberta is unnecessary, as MPB supposedly won't persist in jack pine. However, Figure 8 clearly shows that pine volume also matters. It is also possible that MPB is responding to the availability of large‐diameter trees, and that both pine species identity and large‐diameter tree availability could be strongly correlated due to latent environmental variables not reflected in the pine volume predictor. Such a confounding bias is unlikely, but not impossible.

Our semi‐mechanistic models have several additional limitations. First, our models assume that beetle attack rate parameters are only affected by the forest properties in the destination pixel; in reality, beetles probably respond to forest properties along their flight path. Second, since the simulations operate at the $1{\mathrm{km}}^{2}$ spatial scale, sub‐pixel patchiness in the distribution of pine trees (i.e., forest connectivity) is assumed to not affect MPB dynamics. Third, the model underestimates real‐world spatial autocorrelation of infestations (Figure S2.4), possibly due to clustered dispersal from unpredictable weather. As a consequence, the model is not suitable for analyzing medium‐scale spatial patterns; for example, correlations between patches separated by up to 10 km.

Our study shows that MPB's slow spread through eastern Alberta is mainly due to problems finding and attacking jack pine, rather than issues with reproduction or development. While the amount of pine in an area matters, some inherent properties of jack pine (putatively smaller sizes, thinner phloem, and/or less monoterpenes) seem to be the larger impediment. However, given MPB's extensive impacts, further investigation into this paper's focal contrast—pine species versus pine volume—is warranted. In particular, more detailed data could be utilized to decompose the “effect of pine volume” into the effects of the pine size distribution, pine density, and forest connectivity. Such an analysis would help to clarify the cross‐continental spread risk of this significant forest pest.

## Author Contributions

**Evan C. Johnson:** conceptualization (lead), formal analysis (lead), methodology (lead), software (lead), visualization (lead), writing – original draft (lead), writing – review and editing (equal). **Antonia Musso:** conceptualization (supporting), data curation (equal), writing – review and editing (equal). **Catherine Cullingham:** data curation (equal), funding acquisition (equal), writing – review and editing (equal). **Mark A. Lewis:** funding acquisition (equal), writing – review and editing (equal).

## Conflicts of Interest

The authors declare no conflicts of interest.

## Supporting information


**Data S1:** Supporting Information.

## Data Availability

Code and data are available on figshare (https://doi.org/10.6084/m9.figshare.30359068).
