# Supplementary material for: Biological Barriers to Forest Pest Invasions: A Novel Host Tree Slows Mountain Pine Beetle Range Expansion
Source: Ecol Evol. 2025 Oct 16;15(10):e72296. doi: 10.1002/ece3.72296 (PMC12529056; doi:10.1002/ece3.72296)
Supplement: Supplementary file 1 — Data S1: Supporting Information. [file ECE3-15-e72296-s001.pdf]

Supporting information for "Biological barriers to forest pest invasions: A novel host tree slows mountain pine beetle range expansion"

Evan C. Johnson[1,*], Antonia Musso[2], Catherine Cullingham[3], and Mark A. Lewis[4,5]

[1]*Naos Marine Laboratories, Smithsonian Tropical Research Institute, Ancón, Panama*

[2]*Biological Sciences; University of Alberta; Edmonton, Alberta, Canada*

[3]*Department of Biology; Carleton University; Ottawa, Ontario, Canada*

[4]*Department of Mathematics and Statistics; University of Victoria; Victoria, British Columbia, Canada*

[5]*Department of Biology; University of Victoria; Victoria, British Columbia, Canada*

[*]*Corresponding author: Evan Johnson, JohnsonE@si.edu*

Data sources

Code and data are available on figshare (10.6084/m9.figshare.30359068).

Contents

S1 Model justification and validation **3**

S1.1 Spatial thinning 3

S1.2 Gaussian process model 4

S1.3 Robustness analysis with model #1 5

S1.4 Model #2 with interaction effects 9

S1.5 Model-fitting details 11

S2 Additional tables and figures **12**

S3 The relationship between beetle pressure and brood density **21**

S4 Models and simulations with alternative metrics of forest structure **23**

Appendix S1 Model justification and validation

S1.1 Spatial thinning

Statistical models typically assume that data points are conditionally independent, meaning residuals are uncorrelated across space and time. However, this assumption often fails when working with spatial data due to autocorrelation, where nearby observations are more similar than distant ones.

While spatial autocorrelation can sometimes be explained through predictors (such as dispersal processes or spatially correlated variables like pine volume), residual autocorrelation may persist. This conditional non-independence rarely biases parameter estimates but can make them appear falsely precise, resulting in artificially narrow confidence intervals or posterior distributions.

In our analysis, we investigated this issue by examining spatial autocorrelation in both raw data and model residuals (Fig. S1.1). Although our model accounted for much of the spatial structure in the number of infested trees, some conditional non-independence remained. To address this, we spatially thinned the data by selecting observations at three-kilometer intervals (corresponding to an ”elbow” in the autocorrelation curve) in both the x and y directions, reducing the dataset to one-ninth of its original size. While some autocorrelation remained at 3 km, our fitted model produced marginal posterior distributions comparable to those from an unthinned model with a latent Gaussian process (Fig. S1.2), suggesting our thinning procedure did not lead to overconfident estimates.

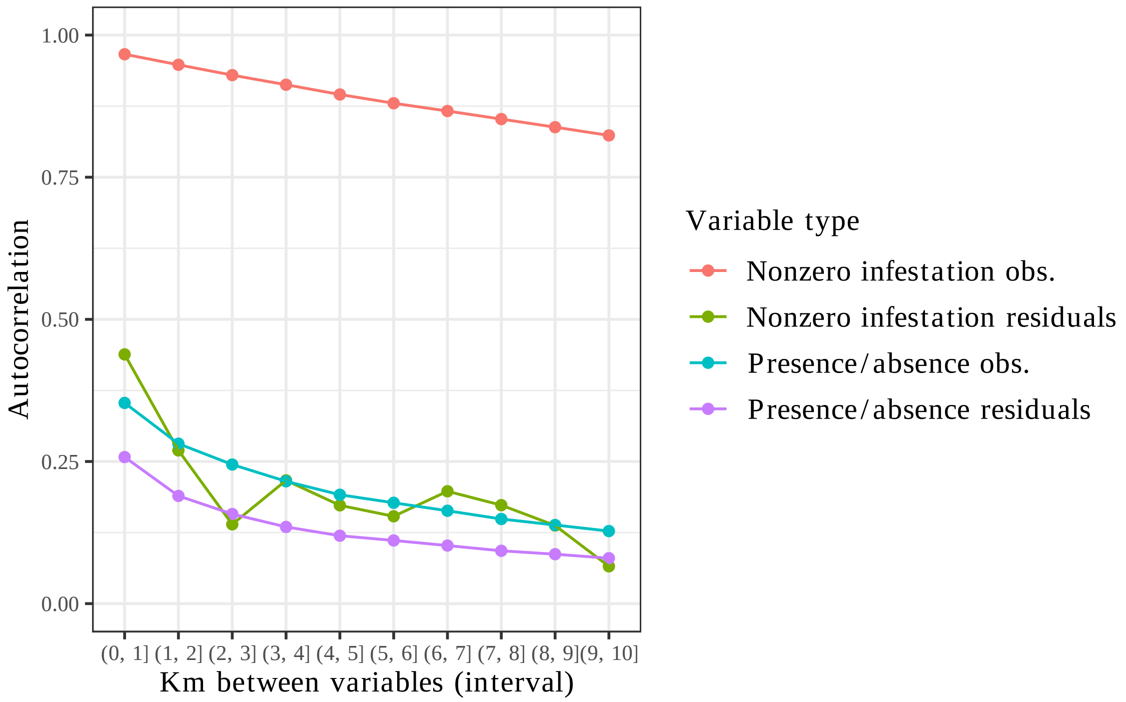

Figure S1.1: Spatial autocorrelations of observations and residuals from model #1 without data thinning. “Nonzero infestations residuals” represent the difference between the logarithm of observed and predicted infestations, for cells where some infestations are present. “Presence/absence residuals” is the difference between the empirical indicator variable (1 for present, 0 for absent) and the predicted probability of presence. Model #1 explains a large fraction of the spatial autocorrelation in the number of non-zero infestations.

S1.2 Gaussian process model

Gaussian process regression, also known as kriging in geography, provides a powerful framework for handling residual spatial autocorrelation. A Gaussian process (GP) is a collection of random variables where any finite subset follows a multivariate normal distribution, completely specified by its mean and covariance functions. Gaussian processes typically model error distributions in multiple regression. In our zero-inflated negative binomial (ZINB) models, the error distribution is not clearly separable, i.e., the variance is jointly determined by the mean and size parameters. Therefore, we utilize a GP as a latent variable affecting the mean parameters of the negative binomial distribution.

The implementation of the Gaussian process modifies our original equations in the main text. For model #1, equation Eq. 5 in the main text becomes:

$$\pi_t(x) = \begin{cases} \operatorname{logit}^{-1}\left(\gamma_{0,L} + \gamma_{1,L} \log\left(B_t(x)\right) + \gamma_{\mathrm{GP}} f_t(x)\right) & \text{if } x \in L, \\ \operatorname{logit}^{-1}\left(\gamma_{0,J} + \gamma_{1,J} \log\left(B_t(x)\right) + \gamma_{\mathrm{GP}} f_t(x)\right) & \text{if } x \in J, \end{cases} \tag{S1.1}$$

Here, γ_{GP} serves as a scale parameter, and f_t represents a year-specific Gaussian process random variable drawn from a multivariate normal distribution with zero mean and covariance function:

$$\Sigma_t(x, y) = \exp\left(-\frac{\mathrm{dist}(x, y)}{2L^2}\right), \tag{S1.2}$$

The Gaussian process similarly affects the count sub-model through a different scaling factor. For model #1, equation Eq. 6 in the main text becomes:

$$f(I_t(x)) = \begin{cases} \mathrm{NB}\left(I_t(x) \mid \mu = \exp\left[\beta_{0,L} + \beta_{1,L}\log\left(B_t(x)\right) + \beta_{\mathrm{GP}} f_t(x)\right], \phi = k_L\right) & \text{if } x \in L, \\ \mathrm{NB}\left(I_t(x) \mid \mu = \exp\left[\beta_{0,J} + \beta_{1,J}\log\left(B_t(x)\right) + \beta_{\mathrm{GP}} f_t(x)\right], \phi = k_J\right) & \text{if } x \in J. \end{cases} \tag{S1.3}$$

Gaussian processes are computationally expensive: fitting a model involves inverting the covariance matrix, generally a $O(n^3)$ operation, where n is the number of pixels under consideration. However, for evenly spaced data — like our projected pixels on a 2D lattice — Fourier transforms can be used to invert the covariance matrix with $O(n \log n)$ operations. Using the *gptools* implementation of this trick (Hoffmann and Onnela, 2023), we were able to fit the model with *Stan*. However, fitting the model was computationally infeasible with 1x1 km pixels, and slow with 5x5 km pixels.

Our ultimate decision to use the non-GP model with spatial data thinning was based on several practical considerations. First, the GP and non-GP model with thinning produced remarkably similar posterior parameter distributions (Fig. S1.2), and this cannot be attributed to the GP model converging to the non-GP model with $L = 0$; instead, the posterior mean of the length-scale parameter was 10 km. Second, the slow fitting of GP models hindered efficient workflow. Finally, the lack of standard methods for calculating marginal likelihoods of zero-inflated negative binomial distributions with GP latent variables complicated our model selection process, specifically when determining whether to group hybrid pines with lodgepole or jack pine in model #1 (see Section 2.2).

S1.3 Robustness analysis with model #1

Previous research and our own experiences suggest that modeling choices can significantly impact scientific inferences (Draper, 1995). A recent statistical analysis of jack pine's suitability, conducted by Xiaoqi Xie et al. (*manuscript in preparation*), found that MPB's "reproduction

rate" (analogous to our effective brood size) was significantly smaller in jack pine. This result prompted us to examine how our modeling decisions affected our conclusions.

We fit six different variants of model #1 in the main text, and found that inferences about the effective brood size face substantial within- and between-model uncertainty, which may alternatively be described respectively as parameter uncertainty and structural uncertainty (Fig. S1.2). On the other hand, all models consistently showed that the effective attack rate is lower in jack pine, as evidenced by $\beta_{0,J}/\beta_{0,L} < 1$. Though other parameters affect the effective attack rate (e.g., $\gamma_{0,J}$, see Section 2.2 for more information) exploratory simulations revealed that the intercept parameters capture the overall pattern of attack rates across pine species.

When applying the same data pre-processing methods as Xie et al. — grouping hybrid and lodgepole pixels without spatial data thinning — we obtained similar results showing significantly lower effective brood size in jack pine. However, this posterior distribution is falsely narrow due to conditional non-independence of the data (Fig. S1.2). Adjusting for residual autocorrelation through spatial data thinning or a Gaussian process latent variable revealed that the effective brood size quotient c_J/c_L has a posterior distribution that substantially overlaps with values above and below 1, with a posterior mean close to unity (see rows 1 and 3 in Fig. S1.2).

When hybrid and jack pine pixels are grouped together, we observe more extreme model uncertainty, with posterior distributions of c_J/c_L that are either completely above or below unity. We compared the hybrid+jack vs. hybrid+lodgepole groupings with approximate leave-one-out cross-validation, as implemented by the *loo* R Package (Vehtari et al., 2019). The expected log predictive density (ELPD) served as the predictive metric. We found that models grouping hybrid and lodgepole pixels performed significantly better according to cross-validation; specifically, $\mathrm{E}[\Delta ELPD] \gg 2 \cdot SE(\Delta ELPD)$. Full details can be found in the file `scripts/model_diagnostics.Rmd` in the supplementary materials. We limited our comparisons to models with spatial data thinning, as models without data thinning would produce artificially narrow $SE(\Delta ELPD)$, and ELPD computation for Gaussian process models is computationally impractical; one would need to use a computationally expensive method to calculate the marginal likelihood (e.g., stepping stone sampling, bridge sampling) for every MCMC sample.

Our robustness analysis reveals substantial structural uncertainty in the effective brood size, but not the effective attack rate. While data pre-processing choices significantly impact

our conclusions, we were able to identify a small subset of good models. Models grouping hybrid and lodgepole pixels show superior predictive performance. Among these, the two models that also account for spatial autocorrelation produced similar posterior distributions, where c_J/c_L was uncertain but close to 1 on average.

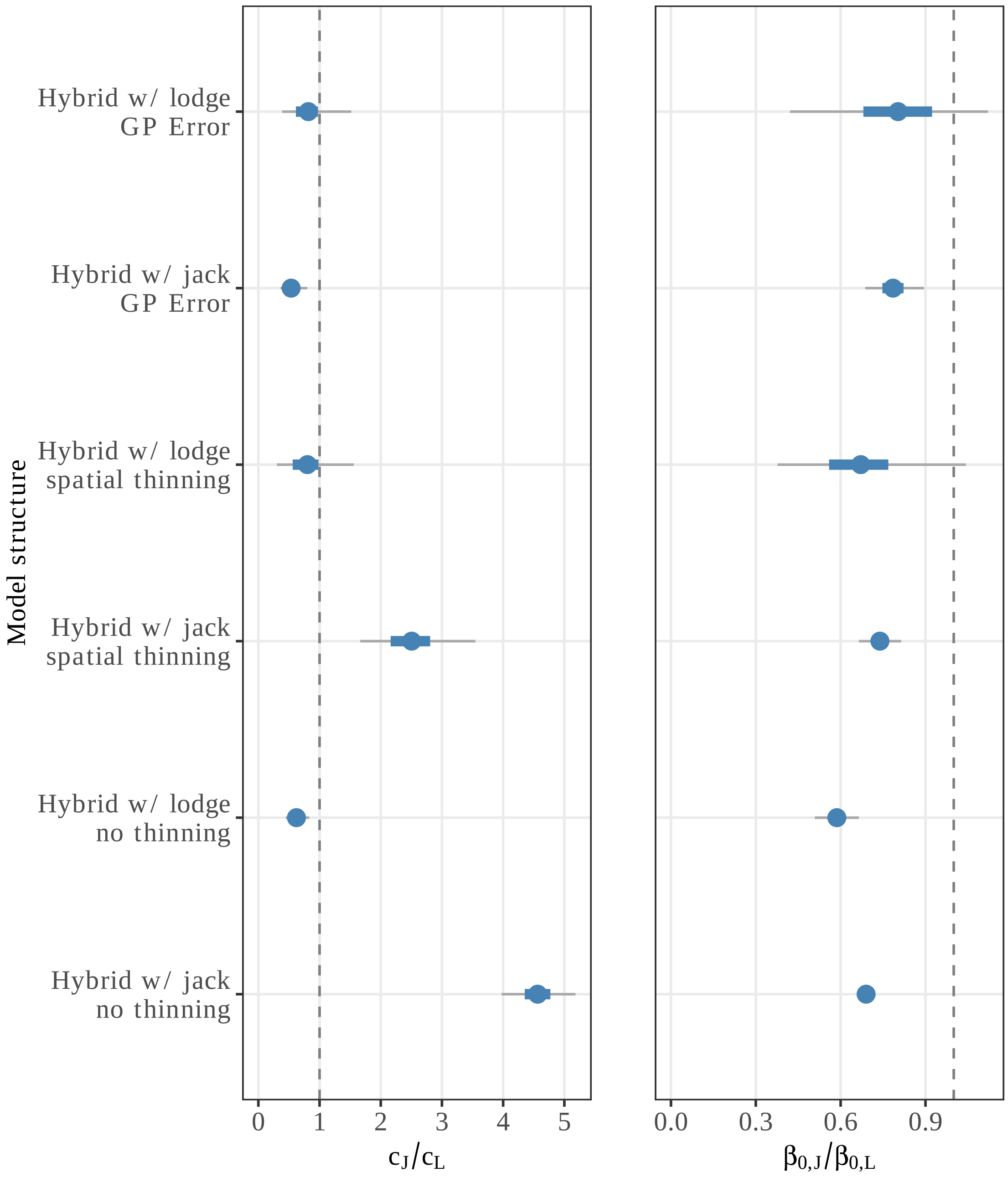

Figure S1.2: There is substantial model uncertainty with respect to the effective brood size, but not the effective attack rate. The points are posterior means, the thick lines are 50% credible intervals, and the thin lines are 95% credible intervals. c_J/c_L below 1 implies that MPB has a smaller effective brood size in jack pine. $\beta_{0,J}/\beta_{0,L}$ below 1 implies that MPB has a smaller effective attack rate in jack pine. "Hybrid with jack" and "Hybrid with lodge" refers to the grouping of hybrid pixels (i.e., $0.1 \leq Q < 0.9$) with pure jack pine or pure lodgepole pine pixels, for the purpose of fitting model #1, as described in Section 2.2 of the main text. "Spatial thinning" refers to the removal of all data except for the intersection of every third row and column, as described in Section 2.1 of the main text. "GP" refers to the Gaussian process models described in Appendix S1.2.

S1.4 Model #2 with interaction effects

We extended Model #2 to include all possible two-way interactions between the predictors. The model structure remains similar but with additional interaction terms in both the presence and count components. The probability of observing a non-zero number of infestations becomes

$$\text{logit}(\pi) = \gamma_0 + \gamma_B B^* + \gamma_Q Q^* + \gamma_V V + \gamma_{BV} B^* V + \gamma_{BQ} B^* Q^* + \gamma_{QV} Q^* V, \tag{S1.4}$$

where B^*, Q^*, and V represent the standardized beetle pressure (log-transformed), pine ancestry, and pine volume (log-transformed) predictors, respectively.

The mean of the negative binomial count distribution similarly becomes

$$\mu = \exp\left[\beta_0 + \beta_B B^* + \beta_Q Q^* + \beta_V V + \beta_{BV} B^* V + \beta_{BQ} B^* Q^* + \beta_{QV} Q^* V\right] \tag{S1.5}$$

Table S1.1 reveals several notable interaction effects. In the presence/absence component, there is a substantial negative interaction between pine ancestry and pine volume ($\gamma_{QV} = -0.42$), suggesting that the effect of pine ancestry on infestation probability decreases in areas with higher pine volume. In the count component, we found smaller positive interactions between beetle pressure and both pine volume ($\beta_{BV} = 0.10$) and pine ancestry ($\beta_{BQ} = 0.15$).

We used counterfactual simulations to show how these interaction effects work together. Figure S1.3 shows these results, which parallel the non-interaction analysis presented in Figure 8 of the main text. These simulations support a main conclusion of this paper: both pine volume and pine ancestry matter, but pine ancestry matters more.

Parameter	Short description	Mean	SD	$CI_{2.5\%}$	$CI_{97.5\%}$
γ_0	Intercept, presence	-3.8	0.083	-4.0	-3.7
γ_B	Beetle pressure, presence	1.6	0.078	1.4	1.7
γ_Q	Pine ancestry, presence	0.32	0.090	0.14	0.50
γ_V	Pine volume, presence	1.2	0.11	0.98	1.4
γ_{BV}	Beetle pressure & pine volume interaction, presence	0.031	0.071	-0.11	0.17
γ_{BQ}	Beetle pressure & pine ancestry interaction, presence	-0.10	0.083	-0.27	0.057
γ_{QV}	Pine ancestry & pine volume interaction, presence	-0.42	0.12	-0.66	-0.18
β_0	Intercept, count	1.8	0.099	1.6	2.0
β_B	Beetle pressure, count	0.037	0.062	-0.087	0.16
β_Q	Pine ancestry, count	0.54	0.12	0.29	0.76
β_V	Pine volume, count	0.010	0.16	-0.31	0.33
β_{BV}	Beetle pressure & pine volume interaction, count	0.10	0.040	0.024	0.18
β_{BQ}	Beetle pressure & pine ancestry interaction, count	0.15	0.068	0.024	0.29
β_{QV}	Pine ancestry & pine volume interaction, count	-0.0045	0.19	-0.37	0.37
k	Dispersion parameter, count	0.42	0.022	0.38	0.47

Table S1.1: Parameter estimates for model #2 with interaction effects. Recall that the predictors B^*, V, and Q^* have been standardized so that the coefficients can be interpreted as predictor importance.

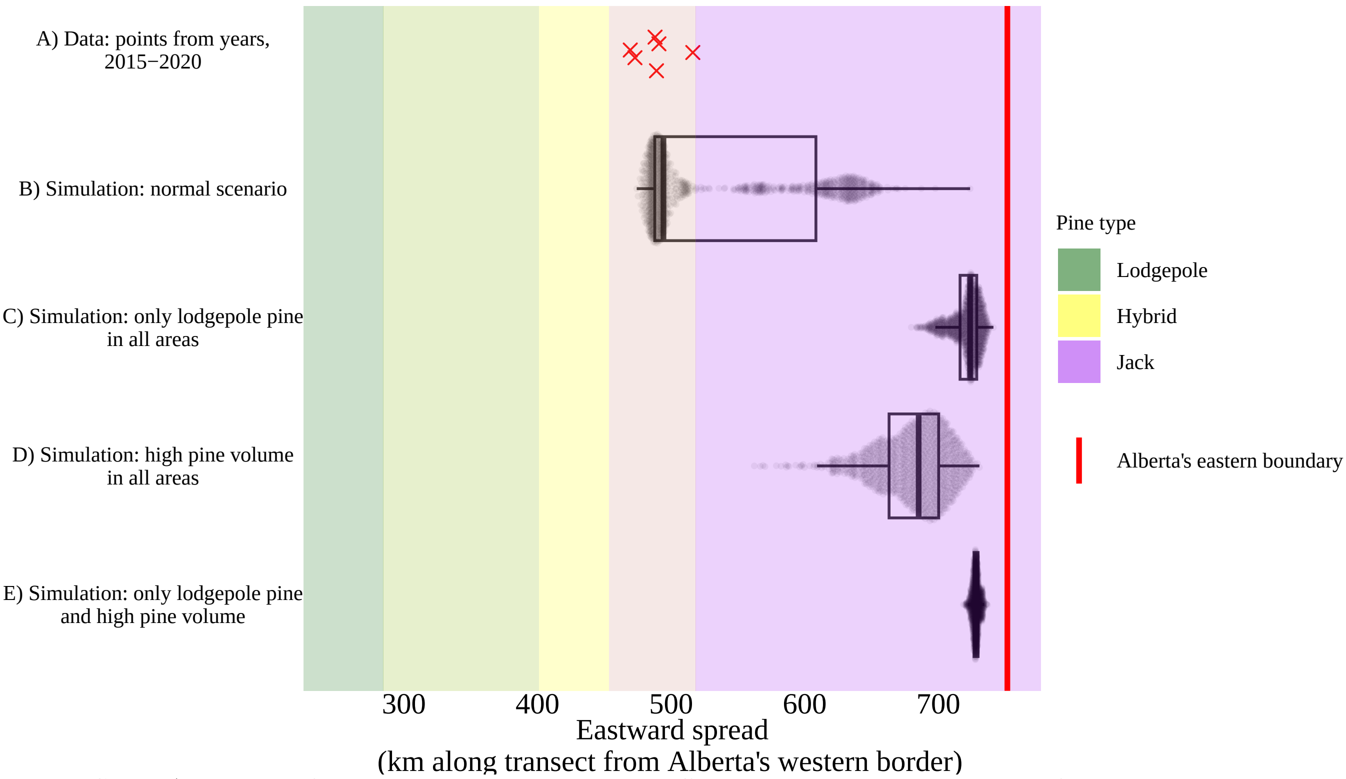

Figure S1.3: A variant of model #2 with interaction effects supports a main result of this paper: mountain pine beetle's slow spread is primarily due to some species-level property of jack pine, and secondarily due to low pine volumes in eastern Alberta. The x-axis shows eastward spread of MPB as measured by the 99th percentile of distances for infestations, from 2015–2020, projected along the projection line (see Fig 5). Overlap in pine type colors represents transition zones between pine types that result from projecting curved 2D species boundaries onto a 1D transect line.

S1.5 Model-fitting details

We examined a standard suite of diagnostics (Gelman et al., 2014, Ch. 6). to ensure that the Markov Chains had converged to a unique posterior distribution and were sampling efficiently. All models under consideration passed these diagnostics successfully. Specifically, we confirmed that $\hat{R} < 1.1$ for all parameters (indicating proper chain mixing), the effective sample size per iteration exceeded 0.001 (demonstrating efficient sampling), the energy Bayesian fraction of missing information (E-BFMI) was below 0.2 (suggesting appropriate model specification), and the proportion of divergent trajectories remained well below 1% (indicating unbiased estimation). The complete diagnostic analysis can be found in the supplementary files, specifically `scripts/model_diagnostics.Rmd and scripts/stan_utility.R`

To evaluate the influence of our prior distributions on the posterior estimates, we calculated the posterior contraction:

$$\text{post. contraction} = 1 - \frac{\mathbb{V}\text{post}}{\mathbb{V}\text{prior}}. \tag{S1.6}$$

This metric quantifies how informative the data are relative to the prior for each parameter. All parameters showed posterior contraction greater than 0.99, with the exception of parameter c_J, which exhibited posterior contraction of 0.83. This is not unexpected, given that the contraction measures the relative informativeness of the data, and the fact that there are few infestations in jack pine. The posterior contraction analysis can also be found in the supplementary files: `scripts/model_diagnostics.Rmd`.

Appendix S2 Additional tables and figures

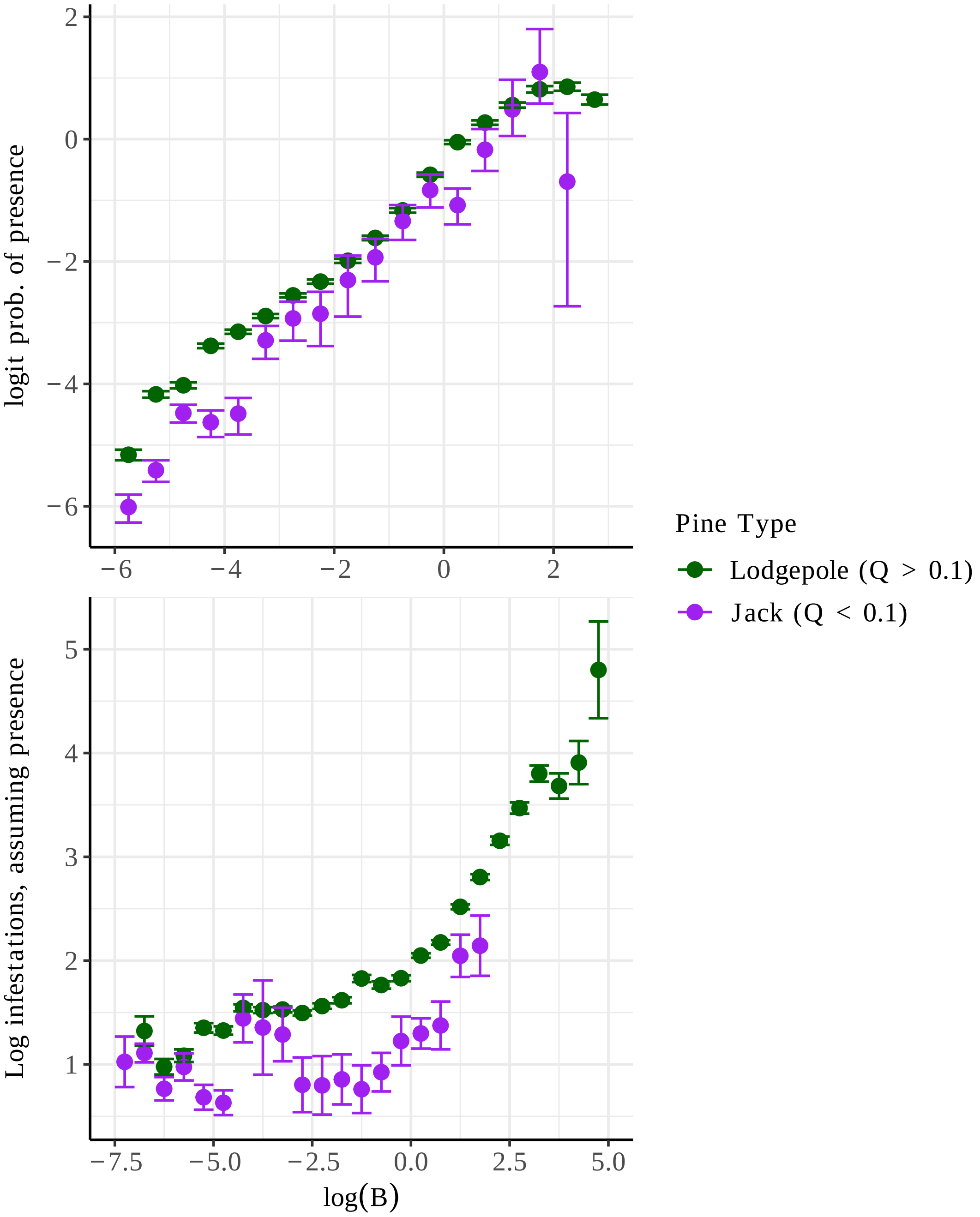

Figure S2.1: Graphical justification of model #1. The logit-scale probability of infestations within a 1×1 km pixel, and the logarithm of total infestations within a 1×1 km pixel (assuming that some infestations are present), are approximately linear with respect to the logarithm of beetle pressure. Points and errorbars show the mean ± 1 standard error across pixels within evenly spaced intervals of log beetle pressure, $\log(B)$.

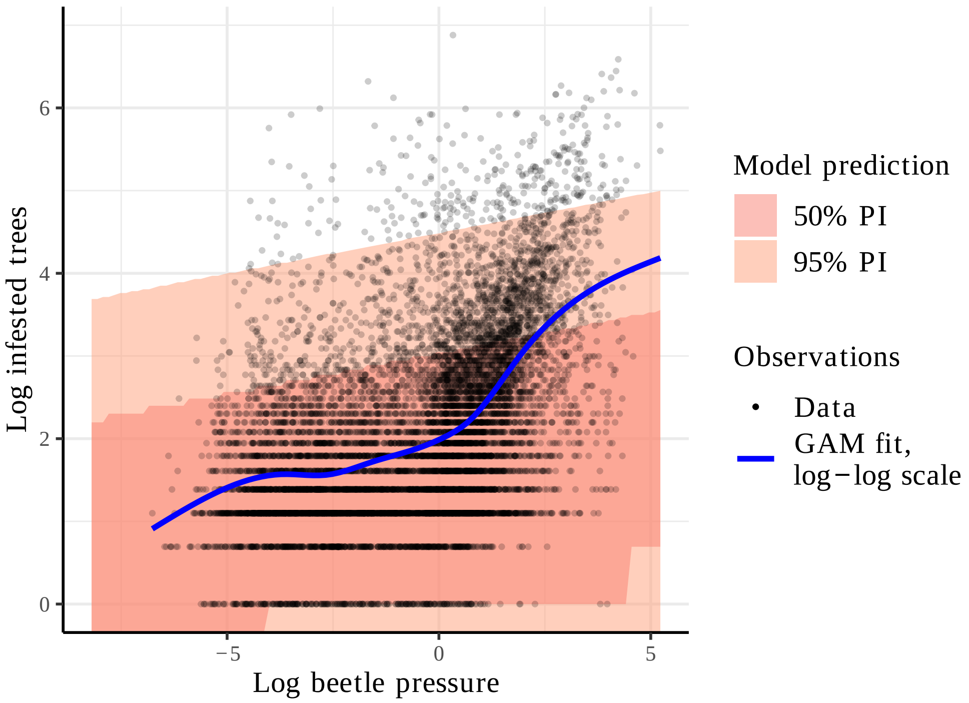

Figure S2.2: Observations and predictive intervals (PIs) for the negative binomial count sub-model of model #1. Each point represents the logarithm of the total number of infested trees within a 1×1 km pixel for a particular year. Pixel-year combinations with zero infestations are not shown.

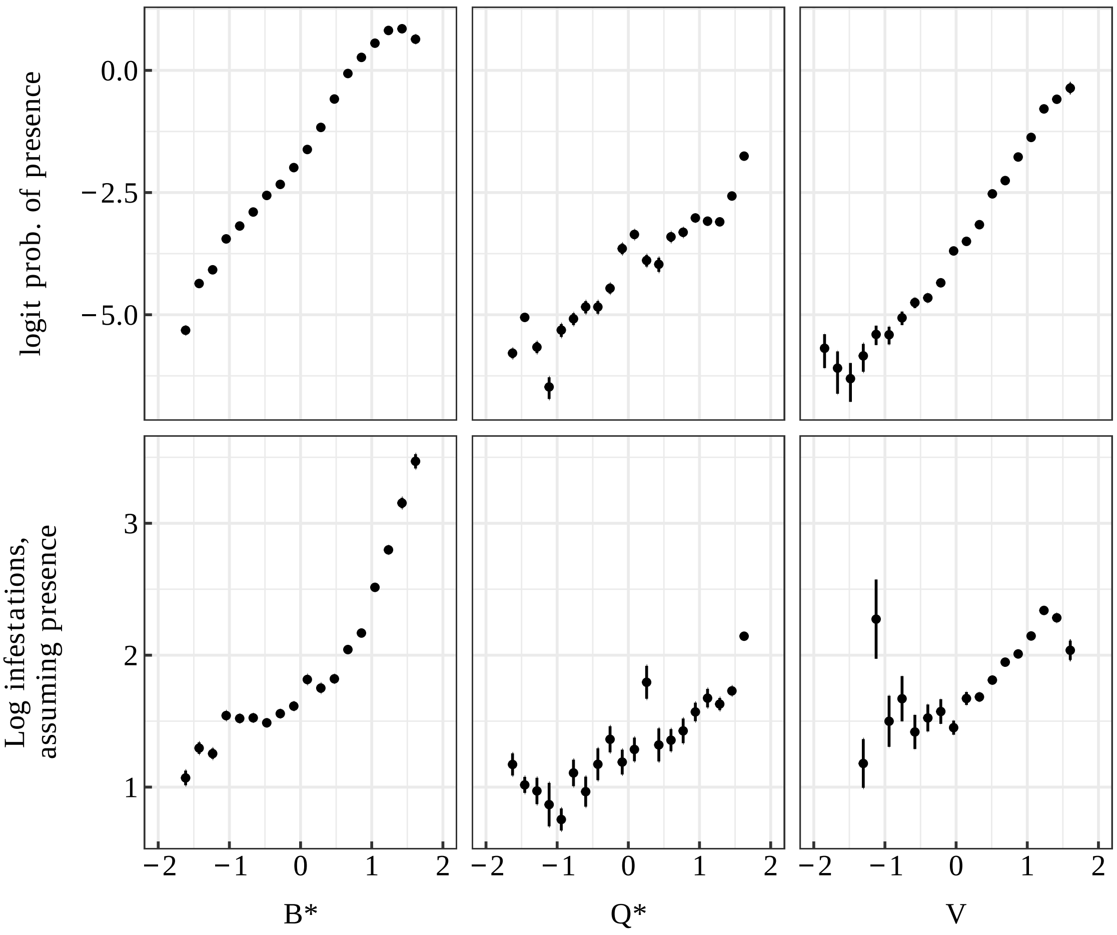

Figure S2.3: Graphical justification of model #2. The logit-scale probability of infestations, and the logarithm of infestations (assuming 1 or more infestations) are approximately linear with respect to all three predictors. Points and errorbars show the mean ± 1 standard error across pixels within evenly spaced intervals of the predictors .

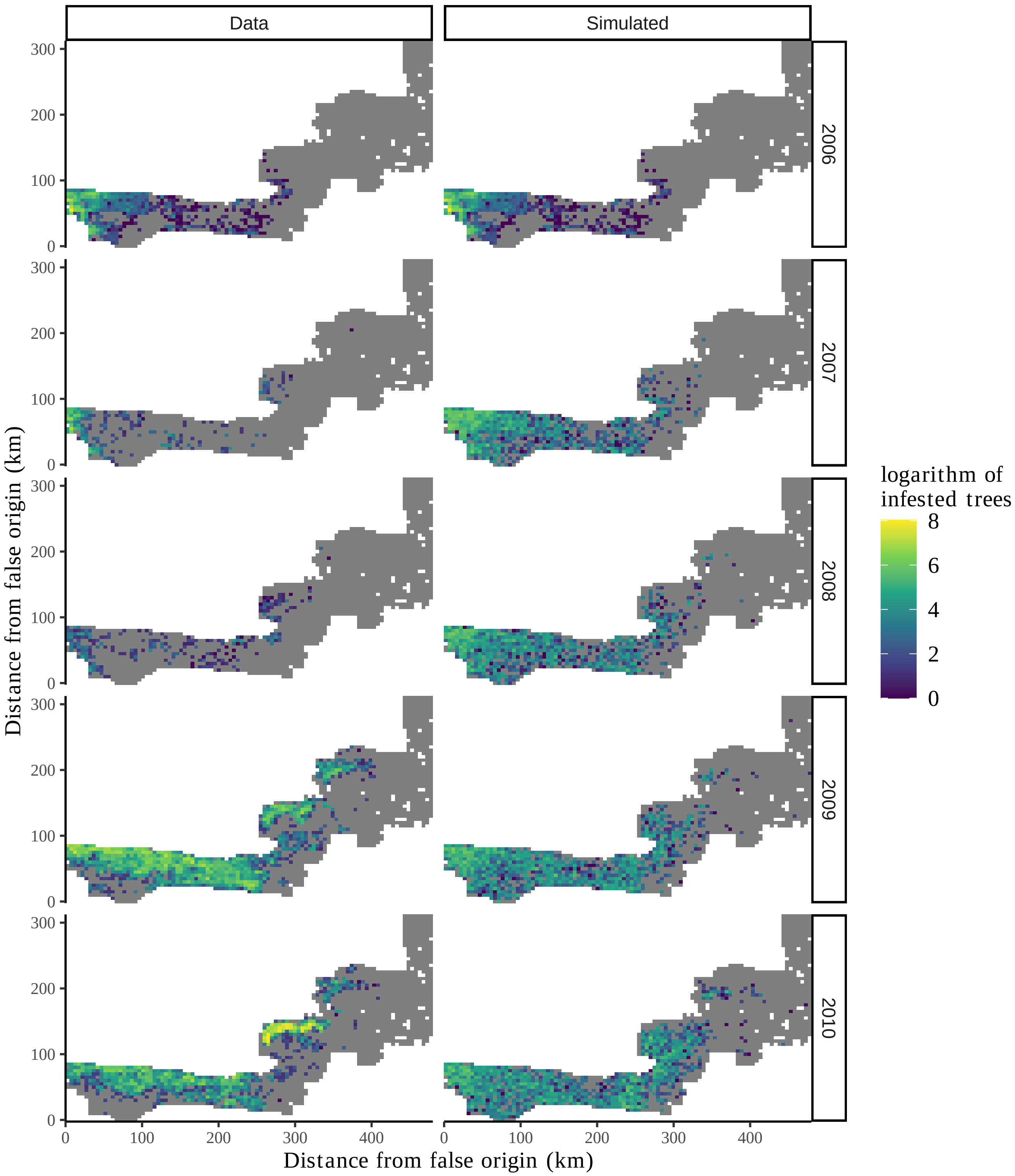

Figure S2.4: Model limitations: simulations of model #1 starting in 2006 do not capture the high number of infestations in 2009 & 2010, nor the patchy distribution of infestations. Data (real and simulated) are shown here in 5x5 km pixels.

Parameter	Short description	Mean	SD	$CI_{2.5\%}$	$CI_{97.5\%}$
c_L	Effective brood size, lodgepole/hybrid	1	0	N/A	N/A
c_J	Effective brood size, jack	0.80	0.33	0.30	1.6
$\gamma_{0,L}$	Presence logit intercept, lodgepole/hybrid	-0.091	0.053	-0.19	0.014
$\gamma_{0,J}$	Presence logit intercept, jack	-1.7	0.47	-2.7	-0.79
$\gamma_{1,L}$	Presence logit slope, lodgepole/hybrid	0.64	0.014	0.62	0.67
$\gamma_{1,J}$	Presence logit slope, jack	0.62	0.086	0.46	0.79
$\beta_{0,L}$	Abundance log intercept, lodgepole/hybrid	2.7	0.037	2.6	2.8
$\beta_{0,J}$	Abundance log intercept, jack	1.8	0.46	1.0	2.8
$\beta_{1,L}$	Abundance log slope, lodgepole/hybrid	0.10	0.0087	0.086	0.12
$\beta_{1,J}$	Abundance log slope, jack	0.074	0.063	0.0023	0.23
k_L	Dispersion param, lodgepole/hybrid	0.40	0.020	0.37	0.45
k_J	Dispersion param, jack	0.67	0.33	0.24	1.5

Table S2.1: Parameter estimates for model #1. Note that the effective brood size in lodgepole pine is fixed at $c_L = 1$ in order for other parameters to be identifiable.

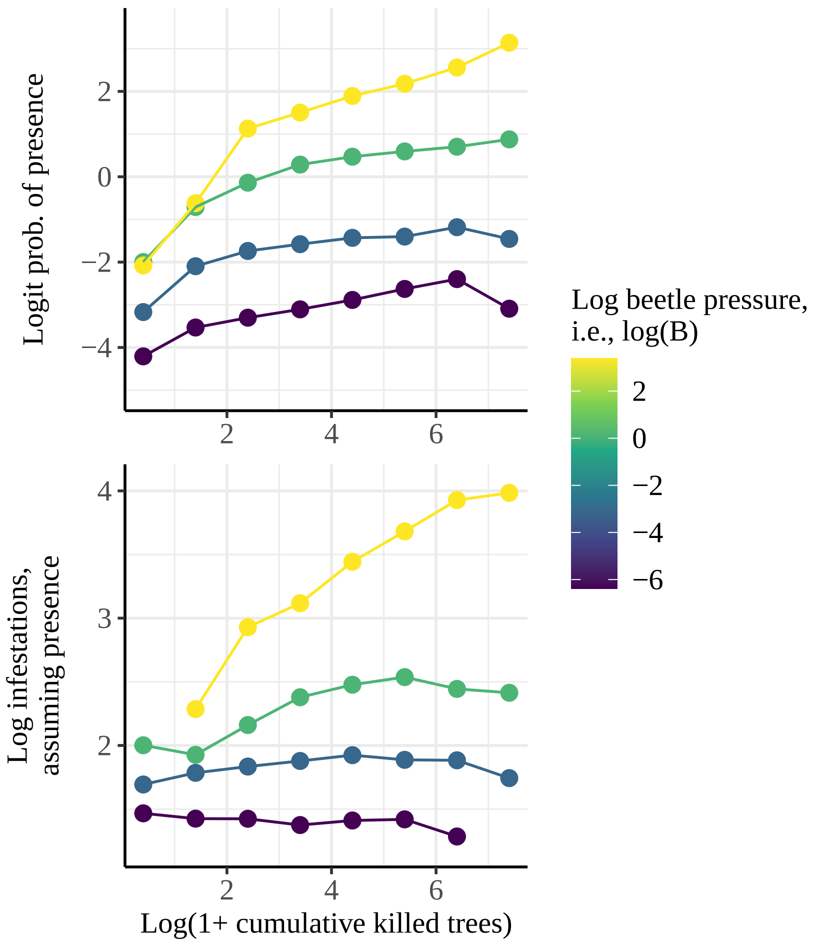

Figure S2.5: There is no clear relationship between infestations next year and the cumulative killed trees. Points represent average estimates within evenly spaced bins across the the natural log of one plus the cumulative killed trees (from 2005 onwards, though only 2009 onward are plotted for consistency with the rest of the analysis) and the natural log of beetle pressure. We stratify by log beetle pressure predictor to avoid detecting a spurious positive correlation between cumulative killed tree and infestations, since areas with higher tree mortality tend to have more beetles and thus higher infestation rates.

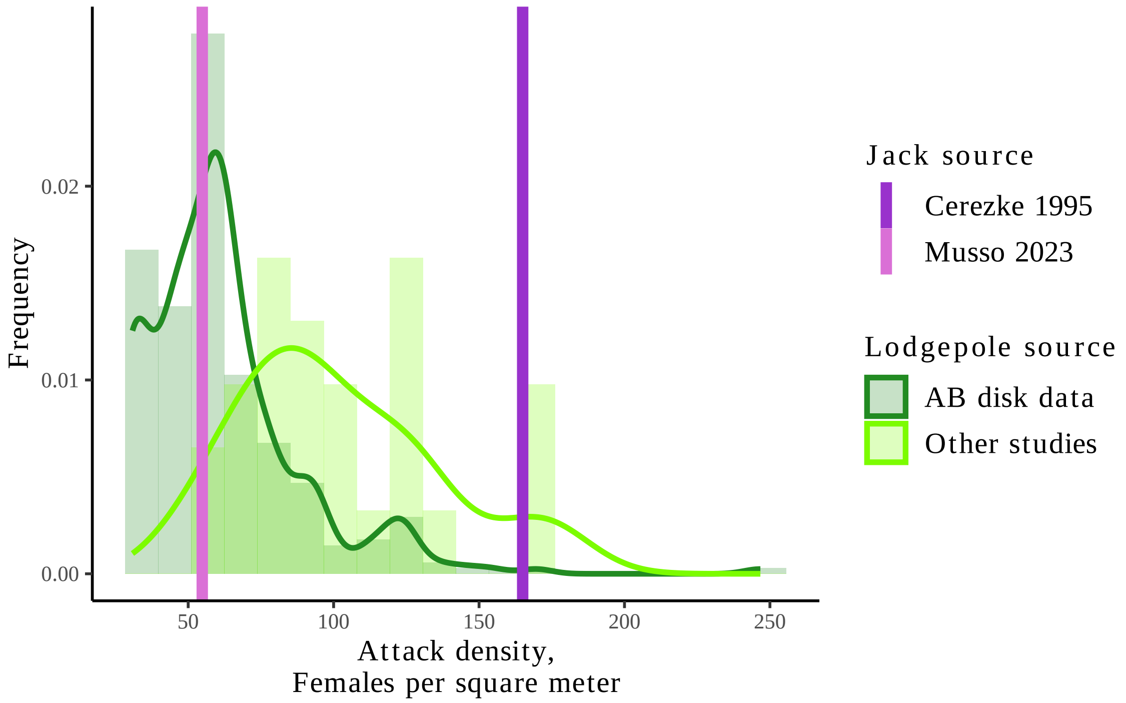

Figure S2.6: Jack pine and lodgepole pine show similar attack densities (measured by entry holes), though jack pine data are limited. The two studies assessing attack density in jack pine (Cerezke (1995) and Musso (2023)) are bolt experiments where bolts or live trees (which were later cut into bolts) were mass attacked by MPB *in situ*. The AB disk data contain entry hole densities estimated from 2 inch diameter disks of bark from infested lodgepole pine across Alberta (Government of Alberta, 2016), and the "Other studies" category includes estimates obtained via entry holes or gallery starts from a number of studies, mostly United States Forest Service reports: Tishmack et al. (2005); Mccambridge (1967); Negrón (2018); Reid (1963); Raffa and Berryman (1983); Rasmussen (1980); Shepard (1965); Knight (1959); De Leon (1939); Safranyik (1988); Safranyik and Vithayasai (1971); Safranyik (1968); Parker (1979); Schmid (1972); Blackman (1931); Whiteside (1937); Washburn and Cole (1959); author N/A (1963); Peterman (1974); Klein et al. (1978); Safranyik and Linton (1991).

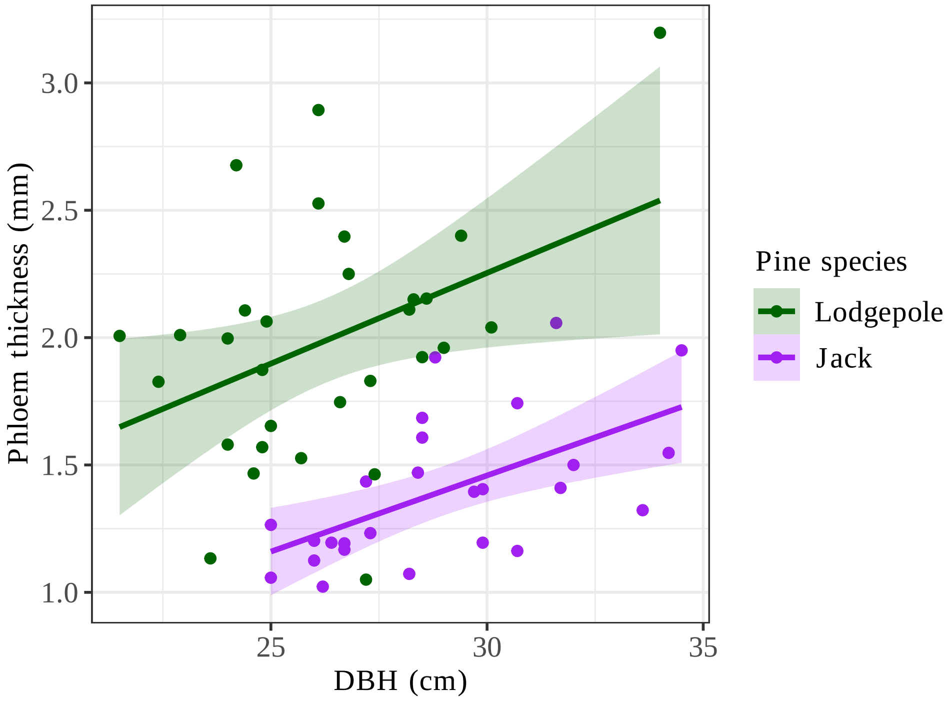

Figure S2.7: Jack pine has thinner phloem than lodgepole pine at the same diameter at breast height (DBH). Data from Musso (2023), chapters 2 & 3.

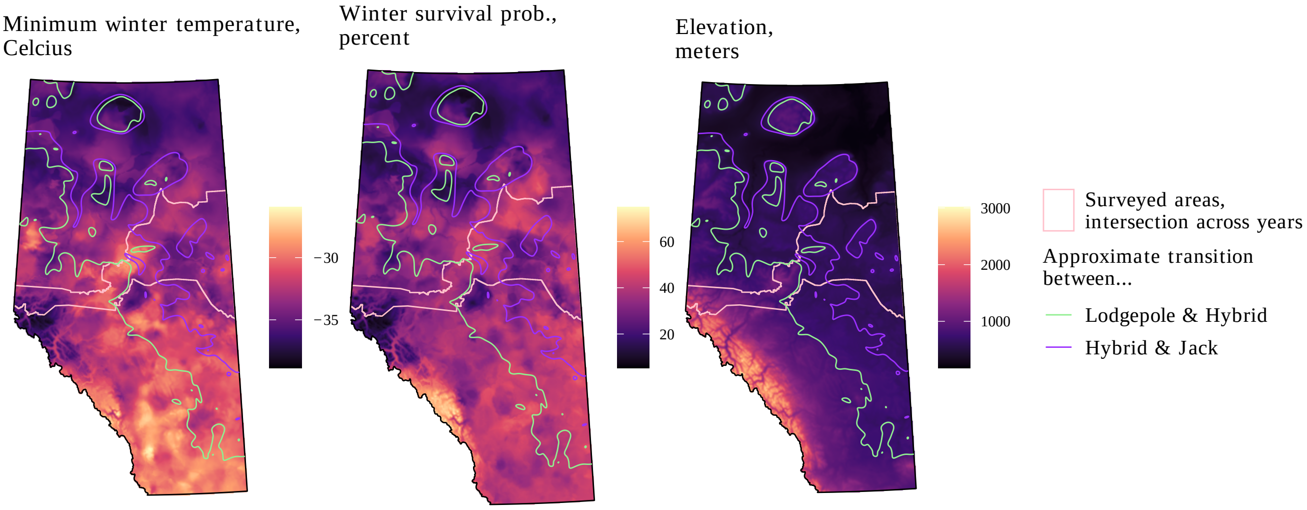

Figure S2.8: The thermal regime in jack pine forests is suitable for MPB development and survival, partly because eastern Alberta has a lower elevation.

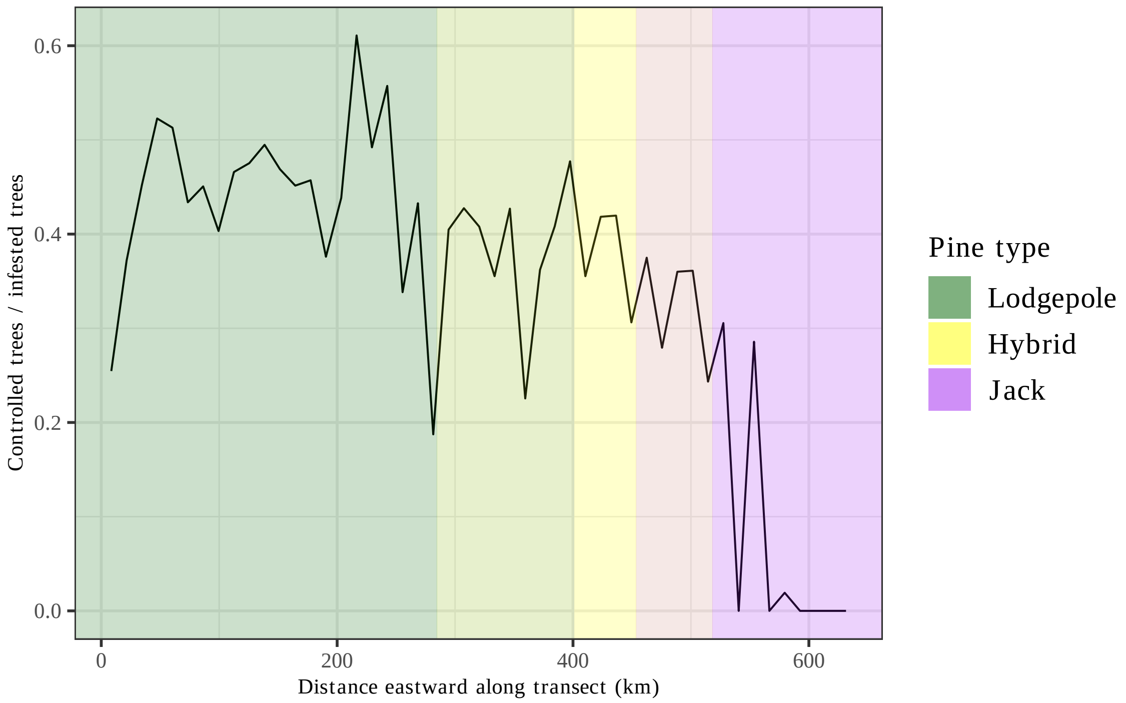

Figure S2.9: A higher proportion of infested trees were controlled in western Alberta, compared to eastern Alberta. This figure was created by projecting infestations and controlled trees within the consistently surveyed area polygon, onto to the projection line, as in Figure 3 in the main text.

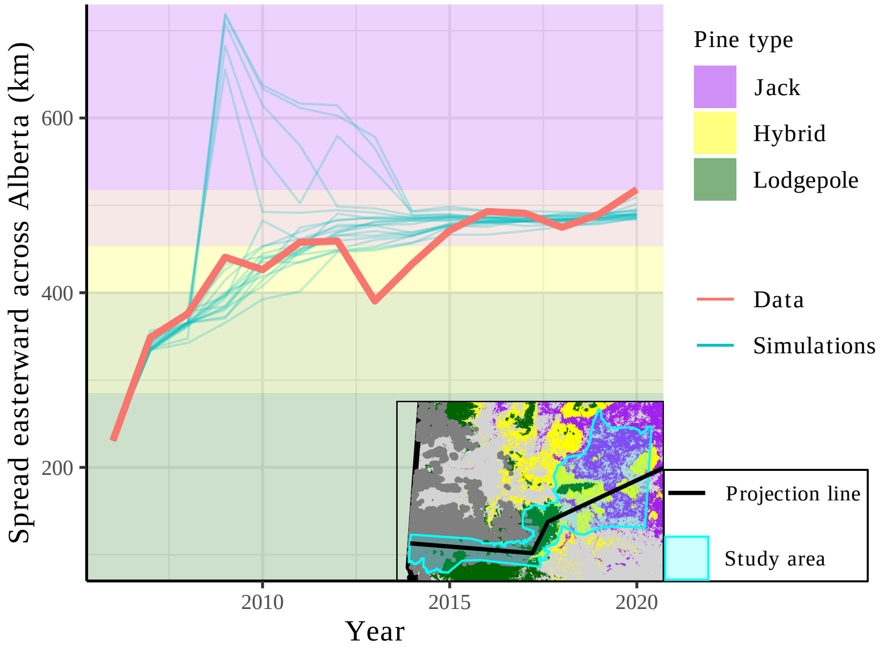

Figure S2.10: Model #1 accurately predicts the observed pattern MPB's decelerating spread. The peak of some simulations in 2009 comes from the fact that the proportion of controlled trees was low in 2009, putatively due to long-distance dispersal from British Columbia into western Alberta (Carroll et al., 2017).

Appendix S3 The relationship between beetle pressure and brood density

To examine the relationship between beetle pressure and brood size, we analyzed Alberta's MPB *disk data* (Government of Alberta, 2016). Collected by Alberta Agriculture and Forestry, this dataset spans from 2008 to 2016, covers over 1000 sites across Alberta, and contains nearly 10,000 trees. The sampling method involves extracting four bark-covered sapwood disks from attacked trees (up to 20 trees per site). Entrance holes and various life stages are counted. Like Goodsman et al. (2018), we excluded trees that contained no larvae or pupae (whether living or dead) from our analysis, since the absence of any life stages indicates these trees were not successfully attacked. The brood size is measured simply as the total number of living MPB (all stages), and brood density per unit of the tree's surface area is easily calculated given that each disk has a 10.6 cm diameter.

As a proxy for beetle pressure, we counted the previous year's infestations within a local neighborhood of each disk sample. For example, if disks are processed during the spring of 2009, then beetle pressure is the number of red-topped trees in the autumn of 2008. To demonstrate that our results are robust to subjective modeling decisions, we utilized circular neighborhoods of two different sizes: one with a 100 m radius, and another with a 500 m radius.

Figure S3.1 shows the lack of a clear relationship between beetle pressure and brood density. We categorized observations into bins based on the number of infestations per hectare, using a bin width of 2. Additional plots using different bin widths (not shown here) revealed similar patterns.

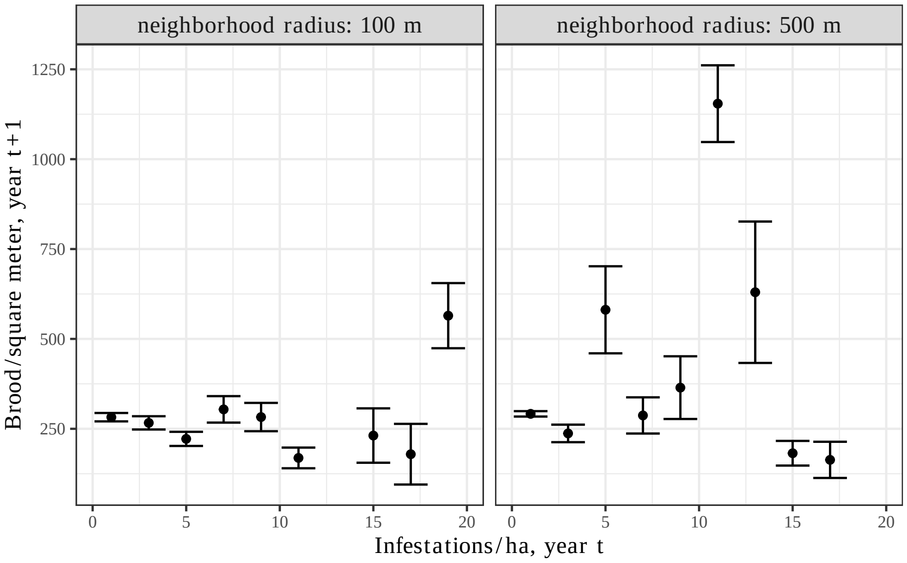

Figure S3.1: The relationship between beetle pressure and brood density, specifically beetles per square meter of tree surface area.

Appendix S4 Models and simulations with alternative metrics of forest structure

Pine volume conflates multiple attributes that could affect MPB population dynamics, including tree density, individual tree size, and canopy closure. Canopy closure putatively influences beetle and pheromone dispersal patterns, and MPB preferentially attacks larger-diameter trees, so disentangling these components of forest structure could provide more nuanced insights into the mechanisms limiting MPB spread. However, our decision to use pine volume to track differences in forest structure between western and eastern Alberta was pragmatic. Pine volume is easily interpretable as the availability of suitable host material. Additionally, the AVIE data is based predominantly on aerial photography interpretation rather than ground-based forest measurements; thus, we lack the tree diameter distributions (or related data) that would be ideal for a more detailed analysis.

Here, we provide a first step toward disentangling forest structural complexity by separating pine cover from habitat quality effects. The first component, pine cover, represents the proportion of each raster cell covered by pine forest. The second component, which we generically call "habitat quality", quantifies how much host material is available per unit of forested area. We operationalized habitat quality using two alternative metrics. Pine volume density is calculated as pine volume divided by pine cover proportion. The Timber Productivity Rating (TPR) serves as an alternative measure, representing a site quality index that measures the potential productivity of an area. For trees $\geq$6 meters tall, TPR is calculated using equations developed for species groups (including deciduous species, white spruce/fir group, pines, and black spruce/tamarack) that project current height and age to what trees would reach at age 50, while for trees $<$6 meters, TPR is professionally interpreted (A.B. Ministry of Agriculture, Forestry and Rural Economic Development, 2022). Homogeneous forested polygons are classified into four productivity classes: Unproductive, Fair, Medium, and Good. We converted this categorical system to a quantitative measure by assigning ordinal values (0, 0.33, 0.66, 1) to these categories and calculating area-weighted averages of forested polygons within each raster cell.

We developed two additional models with identical structure to Model #2, but with decomposed structural predictors. Model #2a includes pine ancestry (Q), pine cover, and pine volume density as predictors, while Model #2b includes pine ancestry (Q), pine cover, and TPR. Both

models used the same zero-inflated negative binomial framework, spatial data thinning, and simulation procedures described in the main text. For counterfactual simulations, we examined scenarios where structural differences between western and eastern Alberta were eliminated by assigning mean values observed in lodgepole pine forests ($Q > 0.9$, pine volume > 1 m^3 km^{-2}) to all pine forest pixels (pine volume > 1 m^3 km^{-2}).

Counterfactual simulations with both models corroborate our main finding: pine species identity is more important than forest structural attributes in limiting MPB spread (Figures S4.1 and S4.2). When pine ancestry effects are eliminated, MPB spreads substantially farther eastward than when any individual forest metric (i.e., pine cover, pine volume density, or TPR) is homogenized. Comparing the relative importance of the two structural components reveals that pine cover and habitat quality contribute approximately equally to limiting MPB spread. For instance, in Model #2a simulations, eliminating differences in pine cover and pine volume density (our first measure of habitat quality) produce similar increases in eastward spread distance. The standardized regression coefficients for pine cover and both habitat quality metrics (not shown here for brevity) have similar magnitudes, again suggesting that the components of forest structure have comparable effects on MPB spread.

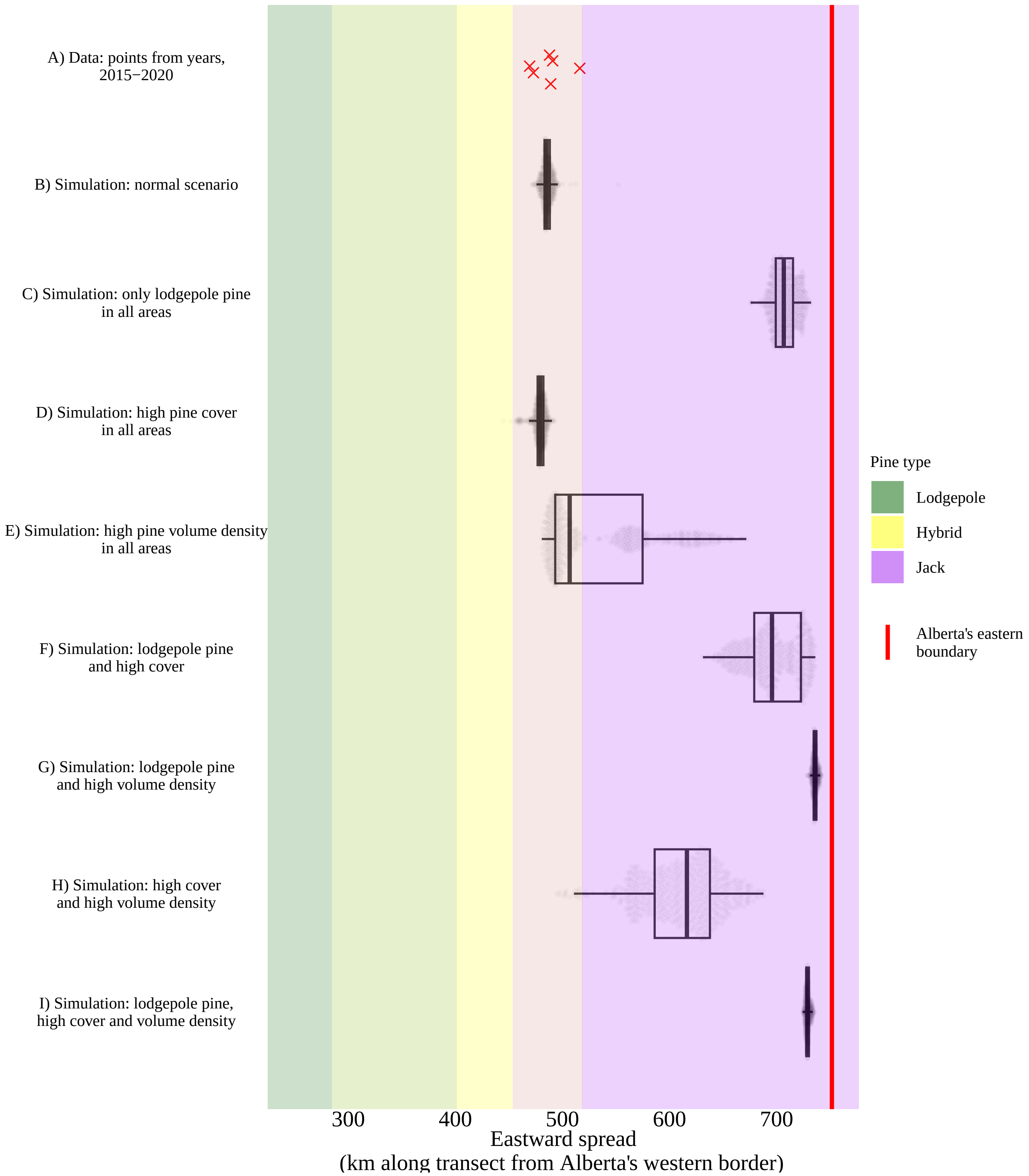

Figure S4.1: Simulation results from model #2a show that pine species identity dominates over forest structural components in limiting MPB spread. Both measures of forest structure, pine cover and pine volume density, have similar effects on spread, though simulations suggest that pine volume density may be slightly more important. The y-axis demarcates the data and different simulation scenarios. The x-axis shows eastward spread of MPB as measured by the 99th percentile of distances for infestations, from 2015–2020, projected along the projection line (see Fig 5). Overlap in pine type colors represents transition zones between pine types that result from projecting curved 2D species boundaries onto a 1D transect line.

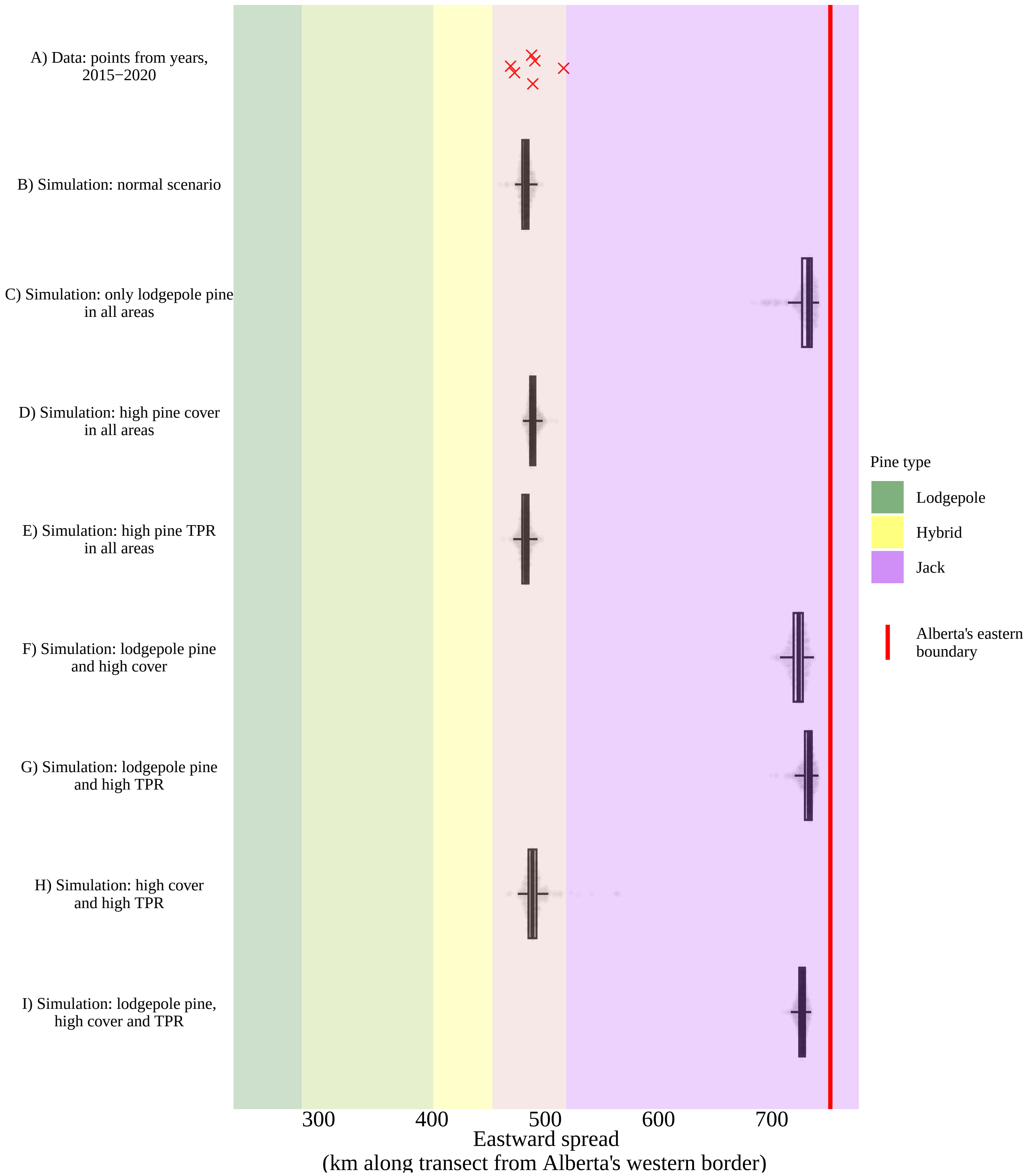

Figure S4.2: Simulation results from model #2b show that pine species identity dominates over forest structural components in limiting MPB spread. Both measures of forest structure, pine cover and Timber Productivity Rating (TPR), have similar effects on spread. The y-axis demarcates the data and different simulation scenarios. The x-axis shows eastward spread of MPB as measured by the 99th percentile of distances for infestations, from 2015–2020, projected along the projection line (see Fig 5). Overlap in pine type colors represents transition zones between pine types that result from projecting curved 2D species boundaries onto a 1D transect line.

References

A.B. Ministry of Agriculture, Forestry and Rural Economic Development (2022). Alberta vegetation inventory standards. version 2.1.5. Technical report, Agriculture, Forestry and Rural Economic Development, Government of Alberta.

author N/A (1963). Mountain pine beetle conditions forest service region 4, november 1963. Technical report, United States Department of Agriculture, Forest Service.

Blackman, M. W. (1931). The black hills beetle. Technical report, Syracuse University, New York State College of Forestry.

Carroll, A., Seely, B., Welham, C., and Nelson, H. (2017). Assessing the effectiveness of Alberta's forest management program against the mountain pine beetle: Final report for fRI research project 246.18 parts 1 and 2. Technical report, fRI Research.

Cerezke, H. (1995). Egg gallery, brood production, and adult characteristics of mountain pine beetle, *Dendroctonus ponderosae* Hopkins (*Coleoptera: Scolytidae*), in three pine hosts. *The Canadian Entomologist*, 127(6):955–965.

De Leon, D. (1939). The biology and control of the black hills beetle (*Dendroctonus ponderosae* Hopk.) summary of studies in Colorado and Wyoming 1935-1938. Technical report, United States Department of Agriculture, Forest Service.

Draper, D. (1995). Assessment and propagation of model uncertainty. *Journal of the Royal Statistical Society: Series B (Methodological)*, 57(1):45–70.

Gelman, A., Carlin, J. B., Stern, H. S., Dunson, D. B., Vehtari, A., and Rubin, D. B. (2014). *Bayesian data analysis.* Chapman and Hall/CRC, 3rd edition.

Goodsman, D. W., Grosklos, G., Aukema, B. H., Whitehouse, C., Bleiker, K. P., McDowell, N. G., Middleton, R. S., and Xu, C. (2018). The effect of warmer winters on the demography of an outbreak insect is hidden by intraspecific competition. *Global Change Biology*, 24(8):3620–3628.

Government of Alberta (2016). Mountain pine beetle detection and management in Alberta. Technical report, Government of Alberta, Agriculture and Forestry. Accessed: 2024-09-05.

Hoffmann, T. and Onnela, J.-P. (2023). Scalable Gaussian process inference with stan. *arXiv preprint arXiv:2301.08836*.

Klein, W. H., Parker, D. L., and Jensen, C. E. (1978). Attack, emergence, and stand depletion trends of the mountain pine beetle in a lodgepole pine stand during an outbreak. *Environmental Entomology*, 7(5):732–737.

Knight, F. B. (1959). Measuring trends of black hills beetle infestations. Technical report, United States Department of Agriculture, Forest Service.

Mccambridge, W. F. (1967). Nature of induced attacks by the black hills beetle, *Dendroctonus ponderosae* (*Coleoptera: Scolytidae*). *Annals of the Entomological Society of America*, 60(5):920–928.

Musso, A. E. (2023). *Pine Wars: A New Host: Interactions between the mountain pine beetle (Dendroctonus ponderosae Hopkins) and its pine hosts in Canada's boreal forest.* PhD thesis, University of Alberta, Department of Biological Sciences.

Negrón, J. F. (2018). Biological aspects of mountain pine beetle in lodgepole pine stands of different densities in Colorado, USA. *Forests*, 10(1):18.

Parker, D. L. (1979). Mountain pine beetle infestation characteristics in ponderosa pine, kaibab plateau, arizona, 1975-1977. Technical report, United States Department of Agriculture, Forest Service, Rocky Mountain Forest and Range Experiment Station.

Peterman, R. M. (1974). *Some aspects of the population dynamics of the mountain pine beetle, Dendroctonus ponderosae in lodgepole pine forests of British Columbia.* PhD thesis, University of British Columbia.

Raffa, K. and Berryman, A. (1983). The role of host plant resistance in the colonization behavior and ecology of bark beetles (*Coleoptera: Scolytidae*). *Ecological monographs*, 53(1):27–49.

Rasmussen, L. A. (1980). Emergence and attack behavior of the mountain pine beetle in lodgepole pine. Technical report, Department of Agriculture, Forest Service, Intermountain Forest and Range.

Reid, R. W. (1963). Biology of the mountain pine beetle, dendroctonus monticolae Hopkins, in the East Kootenay Region of British Columbia: III. Interaction between the beetle and its

host, with emphasis on brood mortality and survival. *The Canadian Entomologist*, 95(3):225–238.

Safranyik, L. (1968). *Development of a technique for sampling mountain pine beetle populations in lodgepole pine.* PhD thesis, University of British Columbia.

Safranyik, L. (1988). Estimating attack and brood totals and densities of the mountain pine beetle in individual lodgepole pine trees. *The Canadian Entomologist*, 120(4):323–331.

Safranyik, L. and Linton, D. (1991). Unseasonably low fall and winter temperatures affecting mountain pine beetle and pine engraver beetle populations and damage in the British Columbia Chilcotin Region. *Journal of the Entomological Society of British Columbia*, 88:17–21.

Safranyik, L. and Vithayasai, C. (1971). Some characteristics of the spatial arrangement of attacks by the mountain pine beetle, *Dendroctonus ponderosae* (*COLEOPTERA: SCOLYTIDAE*), on lodgepole pine1: Appendix: Statistical analysis of the" hole-pairs" experiment. *The Canadian Entomologist*, 103(11):1607–1625.

Schmid, J. (1972). *Emergence, attack densities and seasonal trends of mountain pine beetle (Dendroctonus ponderosae) in the Black Hills*, volume 211. Rocky Mountain Forest and Range Experiment Station, Forest Service, USDA.

Shepard, R. (1965). Distribution of attacks by *Dendroctonus ponderosae* Hopk. on *Pinus contorta* dougl. var. *latifolia Englm. Can. Entomol*, 97:207–215.

Tishmack, J., Mata, S., and Schmid, J. (2005). Mountain pine beetle emergence from lodgepole pine at different elevations near Fraser, CO. Technical report, United States Department of Agriculture, Forest Service, Rocky Mountain Research Station. RMRS-RN-27.

Vehtari, A., Gabry, J., Magnusson, M., Yao, Y., and Gelman, A. (2019). loo: Efficient leave-one-out cross-validation and waic for Bayesian models. R package version 2.2.0.

Washburn, R. I. and Cole, W. E. (1959). Mountain pine beetle and black hills beetle conditions in the pine stands of forest service region 4. Technical report, United States Department of Agriculture, Forest Service.

Whiteside, H. E. (1937). Progress report of a study of the black hills beetle emergence in southeastern Wyoming summer of 1936. Technical report, United States Department of Agriculture, Forest Service.
